# Deception Detection from Five-Channel Wearable EEG on LieWaves: A Reproducible Baseline for Subject-Dependent and Subject-Independent Evaluation

**DOI:** 10.3390/s26031027

**Published:** 2026-02-04

**Authors:** Șerban-Teodor Nicolescu, Felix-Constantin Adochiei, Florin-Ciprian Argatu, Bogdan-Adrian Enache, George-Călin Serițan

**Affiliations:** 1 Doctoral School of Electrical Engineering, Faculty of Electrical Engineering, National University of Science and Technology Politehnica Bucharest (NUSTPB), 060042 Bucharest, Romania; 2 Department of Measurements, Electrical Apparatus and Static Converters, Faculty of Electrical Engineering, National University of Science and Technology Politehnica Bucharest (NUSTPB), 060042 Bucharest, Romania; felix.adochiei@upb.ro (F.-C.A.); florin.argatu@upb.ro (F.-C.A.); bogdan.enache2207@upb.ro (B.-A.E.)

**Keywords:** wearable EEG, low-channel EEG, deception detection, cross-subject classification, subject-dependent classification, deep learning, temporal convolutional networks, discrete wavelet transform

## Abstract

**Highlights:**

**What are the main findings?**

- We evaluate deception detection from five-channel wearable EEG on the public LieWaves dataset (27 subjects, Emotiv Insight headset) under both subject-dependent (same-subject) and subject-independent (cross-subject) protocols.
- In the subject-independent configuration, a compact ResNet-SE model trained on raw overlapping windows and evaluated with a single session-level decision threshold estimated from cross-validated scores attains 66.70% session-level accuracy (AUC = 57.80%) on subjects not seen by the model that predicts them.
- In the subject-dependent configuration, an overlapping short-window Res-TCN-SE-Attention model that fuses raw EEG with DWT-based spectral and handcrafted band-power and Hjorth features reaches 99.94% window-level accuracy on held-out windows from held-out sessions of the same individuals under a heavily overlapping, subject-dependent but session-disjoint split. This near-ceiling window-level figure should be interpreted only as an optimistic upper bound in an autocorrelated, subject-dependent regime, and not as an estimate of deployment-relevant deception-detection ability.

**What are the implications of the main findings?**

- Five-channel wearable EEG thus carries modest but above-chance information for lie–truth discrimination in cross-subject settings, suggesting that portable screening might be technically feasible only in tightly controlled scenarios. At the same time, the pronounced generalization gap between subject-dependent and subject-independent models underscores that substantial additional work on robustness, calibration, and external validation is required before real-world deployment.
- By making our subject-wise splits, windowing rules, decision thresholds, and session-level aggregation explicit, we provide a concrete, reproducible benchmark for five-channel wearable EEG deception detection and highlight the need for standardized, clearly reported evaluation protocols.

**Abstract:**

Deception detection with low-channel wearable EEG requires protocols that generalize across people while remaining practical for portable devices. Using the public LieWaves dataset (27 subjects recorded with a five-channel Emotiv Insight headset), we evaluate to what extent five-channel head-mounted EEG can support lie–truth discrimination under both subject-independent and subject-dependent evaluations. For the subject-independent setting, we train a compact Residual Network with Squeeze-and-Excitation blocks (ResNet-SE) model on raw overlapping windows with focal loss, light data augmentation, and grouped cross-validation by subject; out-of-fold window probabilities are averaged per session and converted to labels using a single decision threshold estimated from the cross-validated session scores. For the subject-dependent setting, we adopt an overlapping short-window Residual Temporal Convolutional Network with Squeeze-and-Excitation and Attention (Res-TCN-SE-Attention) model that fuses raw EEG with discrete wavelet transform (DWT)-based spectral and handcrafted band-power and Hjorth features, using an 80/10/10 split at the recording/session level (stratified by session label), so that all windows from a given session are assigned to a single subset; because each subject contributes two sessions, the same subject may still appear across subsets via different sessions. The subject-independent model attains 66.70% session-level accuracy with an AUC of 0.58 on unseen subjects, underscoring the difficulty of person-independent generalization from low-channel wearable EEG. Because practical deployment requires generalization to previously unseen individuals, we treat the subject-independent evaluation as the primary estimate of real-world generalization. In contrast, the subject-dependent pipeline reaches 99.94% window-level accuracy under the overlapping sliding-window (OSW) setting with a session-disjoint split (no session contributes windows to more than one subset). This near-ceiling performance reflects the optimistic nature of subject-dependent evaluation with highly overlapping windows, even when avoiding within-session train–test overlap, and should not be interpreted as a meaningful indicator of deception-detection capability under realistic deployment constraints. These results suggest limited, above-chance separability between lie and truth sessions in LieWaves using a five-channel wearable EEG under the studied protocol; however, performance remains far from deployment-ready and is strongly shaped by evaluation design. Explicit reporting of both protocols, together with clear rules for windowing, aggregation, and threshold selection, supports more reproducible and comparable benchmarking.

## 1. Introduction

Deception detection remains a long-standing scientific and societal challenge with implications for forensic assessment, security screening, and human–computer interaction [1,2]. Conventional polygraph protocols that monitor peripheral physiology (e.g., electrodermal activity, respiration) have been repeatedly criticized for limited validity and susceptibility to countermeasures [3], motivating interest in brain-based alternatives [4,5,6]. Electroencephalography (EEG) offers non-invasive, millisecond-level access to large-scale cortical dynamics [7] and has been used to probe cognitive operations such as memory, attention, and cognitive control [8,9] during truthful responding and concealment [10,11,12]. Beyond classical event-related potential (ERP) markers, time–frequency analyses have reported task-dependent modulations, including delta/theta power increases and alpha/beta power decreases over fronto-central and parietal regions during deception, with gamma-band changes described more variably across paradigms [13,14]. Other studies have examined inter-trial phase consistency, cross-frequency interactions, and connectivity within fronto-parietal control and salience networks [15,16]. Overall, the literature suggests that multivariate combinations of band-power, spectral ratios, time–frequency, and statistical descriptors can offer richer information than ERP-only pipelines for deception-related EEG decoding [6,17,18], even though many of these markers are sensitive to task design and individual variability.

Two broad methodological strands can be distinguished in EEG-based deception research. The first centers on event-related paradigms, particularly Concealed Information Test (CIT) designs, where cognitive ERP components (e.g., P300) elicited by probe stimuli matching concealed knowledge are contrasted with responses to neutral stimuli [19]. Although the forensic literature often discusses deception paradigms alongside the Comparison Question Test (CQT), CQT refers to a questioning protocol, whereas the present distinction is methodological—ERP/CIT-style markers versus oscillatory/time–frequency EEG features—independent of the interrogation protocol. While robust effects are often observed at the group level, the stability of such markers across individuals, tasks, and real-world contexts remains debated [10,20]. A complementary line of work emphasizes oscillatory markers and time–frequency features coupled with machine learning; in this setting, band-power descriptors, Hjorth parameters, and spectral ratios are commonly used characteristics that yield competitive subject-dependent performance in controlled studies [21,22]. Across both strands, a recurring difficulty is generalization: performance that appears high under subject-dependent evaluation (where windows from the same person appear in both training and test sets) can drop markedly when moving to subject-independent evaluation that reserves entire subjects for testing [23,24,25,26]. In addition, heavy temporal overlap between training and test windows can further inflate subject-dependent metrics. This non-stationarity and subject specificity have motivated transfer-learning and domain-alignment methods in related EEG applications [27,28,29], but their impact on deception detection with low-channel, wearable setups is still poorly understood.

More broadly, recent EEG decoding research increasingly emphasizes generalization across subjects, tasks, and recording conditions, rather than optimizing for a single dataset split or subject-specific setting. This trend is reflected in emerging community benchmarks and challenges that explicitly target cross-subject and cross-task transfer, encouraging models that learn representations robust to inter-individual variability and protocol differences [30]. At the modeling level, transformer-based architectures have gained traction for capturing longer-range temporal dependencies in EEG and for supporting end-to-end learning pipelines across multiple EEG application domains [31,32,33]. In parallel, connectivity-aware deep-learning increasingly represents EEG channels as graphs and applies attention-based or spatio-temporal graph models to exploit inter-channel interactions, often using functional connectivity measures such as mutual information or normalized mutual information to construct the underlying connectivity structure [34,35]. Finally, self-supervised and contrastive learning are being actively explored to reduce reliance on labeled data and to promote more subject-invariant representations that improve cross-subject robustness [36,37]. These developments motivate the need for clearly specified, protocol-aware evaluation when assessing EEG-based deception detection, especially for low-channel wearable recordings, so that reported gains reflect genuine generalization rather than favorable splits or subject-dependent effects [38,39].

Representative end-to-end EEG pipelines illustrate both the promise and the practical constraints of moving beyond handcrafted feature engineering. For example, Chen et al. propose an end-to-end graph-attention/convolutional neural network (CNN) framework that inputs preprocessed EEG together with a mutual-information (MI)-derived adjacency matrix to explicitly model inter-channel interactions, reporting cross-subject validation and robustness analyses in a fatigue-detection context [40]. Broader reviews in adjacent EEG applications (e.g., emotion recognition) similarly emphasize that CNN/recurrent neural network (RNN)/graph neural network (GNN)/transformer-based architectures can learn spatio-temporal representations directly from EEG, but note that generalization, interpretability, and protocol sensitivity remain recurring limitations, often amplified by small or heterogeneous datasets [41]. Importantly, information-theoretic connectivity measures such as MI/normalized MI (NMI), and functional connectivity inference more generally, can be sensitive to estimator choice and to acquisition and preprocessing decisions [42], considerations that become more acute for short sliding windows and low-channel wearable montages with limited spatial sampling. Within deception detection, end-to-end models (including graph-convolutional and deep recurrent variants) demonstrate feasibility, but reported performance remains tightly coupled to dataset characteristics and evaluation protocol [43,44,45]. Accordingly, we position our contribution as a protocol-aware, reproducible benchmark for five-channel wearable EEG deception detection, contrasting feature-based and end-to-end baselines under explicitly defined windowing, aggregation, and thresholding procedures, rather than as an application-specific attempt to optimize a single end-to-end architecture.

In parallel to end-to-end representation learning, a longstanding line of work adopts feature-driven information-theoretic modeling, where MI or NMI is used to quantify nonlinear statistical dependence between EEG channels (or regions) and to construct connectivity-derived feature representations. For example, recent work on driving-state decoding computes band-specific NMI-based functional connectivity matrices and uses the resulting NMI features with a hyperparameter self-optimized Gaussian-kernel radial-basis-function extreme learning machine (RBF-ELM), illustrating how MI/NMI can serve as an informative engineered descriptor for EEG classification [46]. In deception-related EEG settings, MI-based connectivity has likewise been explored to characterize functional network differences between truthful and deceptive responses [47]. At the same time, information-theoretic connectivity estimates can be sensitive to estimator choice, window length, and preprocessing decisions, which is especially consequential for short-window analyses and low-channel wearable montages with limited spatial sampling [42,48]. In contrast to MI/NMI feature pipelines that explicitly estimate functional connectivity, the present study benchmarks compact models using raw windows and hybrid representations (raw + DWT and spectral–statistical features), with a primary emphasis on protocol-aware evaluation and session-level decision-making to assess generalization under realistic cross-subject testing.

Against this methodological backdrop, wearable low-channel EEG has emerged as a practical avenue for extending cognitive monitoring outside laboratory environments [49,50,51]. Head-mounted systems with 5–8 channels, such as devices sampling from frontal and temporal sites (e.g., AF3, T7, Pz, T8, and AF4), reduce setup time and cost and avoid the logistics of dense caps, which is attractive for fieldable deception assessment. These advantages come with trade-offs: limited spatial sampling, reliance on dry or semi-dry electrodes that are more sensitive to motion and interface artifacts, and tighter constraints on preprocessing and model complexity than in high-density, gel-based recordings [52,53,54,55,56]. Reviews of wearable EEG emphasize the need for transparent signal-processing choices (e.g., band pass and notch filtering), robust yet simple artifact handling, and clear reporting of evaluation protocols to enable meaningful comparison across studies [57,58,59,60]. In this context, publicly available datasets acquired with head-mounted devices are particularly valuable, because they allow fully reproducible baselines and make performance bounds under realistic constraints more visible [58]. The LieWaves dataset is one such resource: it comprises recordings from 27 participants, each contributing two approximately 75 s sessions (truth and deception) acquired with a five-channel head-mounted system under instructed truth-telling and deception in response to visual stimuli, and has been used in recent work on low-channel deception-related EEG [61,62].

Against this background, we examine deception detection from five-channel wearable EEG on the LieWaves dataset under two complementary evaluation protocols. Subject-independent (cross-subject) evaluation approximates a realistic deployment scenario in which the model is applied to previously unseen individuals. Subject-dependent evaluation, by design, allows the same individuals to appear across subsets and therefore provides an optimistic reference. If overlapping windows are additionally partitioned using a window-level split, temporal dependence between adjacent windows can confound performance estimates (e.g., when near-adjacent windows are assigned to different subsets); this confound is avoided when partitioning is performed at the recording/session level before windowing. Our primary aim is, therefore, not to propose a single definitive model, but to illustrate how much reported performance can change with the evaluation protocol when using architectures that are computationally lightweight and, in principle, compatible with deployment on portable EEG hardware, even though we do not implement, and do not claim to achieve, on-device deployment here.

Concretely, we address two questions: (i) to what extent a compact cross-subject model trained on raw five-channel wearable EEG can distinguish instructed deception from truth on subjects not seen during training; and (ii) how large do the apparent performance gains become when using heavily overlapping, subject-dependent window-level evaluation with richer feature sets? To this end, we (i) train a Residual Network with Squeeze-and-Excitation blocks (ResNet-SE) on raw overlapping 3.0 s windows with focal loss, light data augmentation, and grouped K-fold cross-validation (CV) by subject, aggregating window probabilities into session-level scores and converting them to labels using a single decision threshold estimated from cross-validated out-of-fold session predictions; and we (ii) train an overlapping 2.0 s/0.25 s short-window Residual Temporal Convolutional Network with Squeeze-and-Excitation and Attention (Res-TCN-SE-Attention) model in a subject-dependent setting on a stratified 80/10/10 split at the recording/session level (i.e., all windows from a given session are assigned to a single subset), fusing raw EEG with discrete wavelet transform (DWT)-based and classical spectral–statistical features. Reporting these two pipelines side by side allows us to contrast a realistic cross-subject baseline with the near-ceiling performance typically observed under subject-dependent, highly overlapping windowing, even when sessions are kept disjointed across splits. Throughout, we treat the cross-subject protocol as the main indicator of generalization to new users and present the subject-dependent overlapping sliding-window (OSW) results as an optimistic subject-dependent reference. Taken together, the goal is to provide a clear reference point for future work on deception detection from low-channel wearable EEG, and to make the practical consequences of evaluation protocol choices visible. Specifically, this study contributes:

- A protocol-aware benchmark on the LieWaves five-channel wearable EEG dataset, reporting performance under both subject-dependent OSW and subject-independent cross-subject evaluation.
- A compact cross-subject deep-learning baseline (ResNet-SE) with subject-wise grouped cross-validation and session-level decision-making via window-probability aggregation and a single receiver operating characteristic (ROC)-based threshold estimated from cross-validated session scores.
- A subject-dependent short-window pipeline (Res-TCN-SE-Attention) combining raw windows with wavelet-derived and spectral/statistical features, evaluated under an OSW setting with a single fixed stratified 80/10/10 split at the recording/session level (i.e., all windows from a session inherit its subset assignment); because each participant contributes two sessions, the same participant may still appear across subsets via different sessions.
- Fully specified methodological choices (windowing, splitting, aggregation, and thresholding), together with classical/CNN baselines, to support reproducible comparison under wearable constraints.

## 2. Materials and Methods

### 2.1. Datasets and Selection Rationale

We considered four publicly available EEG datasets that include deception-related paradigms. Their core characteristics, number of EEG channels (Ch), sampling rate (f_s,_ in Hz), number of participants (N), paradigm, and access, are summarized in Table 1. All analyses in this work are conducted on the LieWaves dataset; the remaining datasets are described for context and to situate our results within the broader landscape.

For the present experiments, we selected LieWaves for three main reasons. First, it matches the wearable, low-channel constraint that motivates our focus on practical, out-of-lab use, providing five head-mounted channels sampled at 128 Hz from 27 participants. Second, its session structure is simple and standardized: each participant completes two approximately 75 s sessions, one under instructed truth-telling and one under instructed deception in response to visual stimuli. This facilitates reproducible segmentation into analysis windows and clear session-level aggregation. Third, the data are distributed in a straightforward raw CSV format with balanced truth/lie labels and are easily obtainable, which supports transparent pipelines and independent replication [62].

The other datasets are valuable but align less directly with our specific goals. Deception_data is a high-density, 128-channel, 3000 Hz dataset built around a multi-condition deception card game with spontaneous truthful/false claims and a richer trial taxonomy [63], which would require additional design choices to adapt to continuous session-level detection. Bag-of-Lies contains naturalistic, multimodal recordings (video, audio, and gaze) with the EEG released publicly for a subset of participants, and the full set available on request [64], making exact replication more dependent on author-mediated access. The Dryad dataset focuses on concealed-information-style ERP paradigms (e.g., P300 in guilty/innocent groups) rather than continuous deception versus truth at the session level [65]. In principle, channels from these datasets could be down-selected to mimic a low-channel montage, but their paradigms and hardware are less aligned with our aim of establishing a transparent, reproducible baseline for deception detection using a five-channel wearable system.

### 2.2. Study Workflow

**Figure 1:**
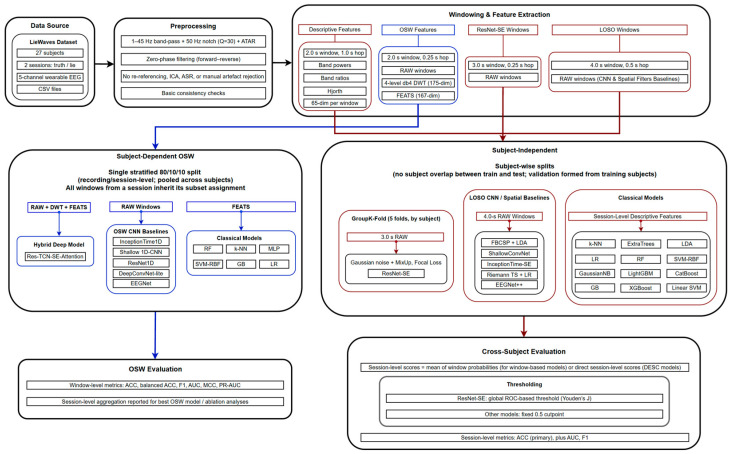
Study workflow. LieWaves recordings (27 subjects, two sessions each) undergo a common preprocessing pipeline (50 Hz notch, 1–45 Hz band pass, zero-phase filtering, and automatic wavelet-packet artifact reduction (ATAR); no re-referencing, ICA, or manual artifact rejection) and are then segmented into several window configurations. Given the 45 Hz upper cutoff, the 50 Hz notch has negligible practical impact here and is included mainly for preprocessing consistency. Two-second windows support descriptive band-power features and the subject-dependent overlapping sliding-window (OSW) branch with RAW, DWT, and FEATS inputs; 3.0 s windows feed the grouped cross-validation Residual Network with Squeeze-and-Excitation blocks (ResNet-SE) model for subject-independent analysis, and 4.0 s windows feed the leave-one-subject-out (LOSO) CNN and spatial-filter baselines. Hand-crafted features are used by classical models and as auxiliary inputs to the hybrid OSW architecture, while raw windows feed convolutional networks. In the OSW branch, recordings are first partitioned (80/10/10) into train/validation/test sets, and overlapping windows are then generated within each partition. Because each participant contributes more than one recording, this remains a subject-dependent setting, while ensuring that each recording is evaluated as a coherent unit. In the subject-independent branch, grouped cross-validation or LOSO splits enforce subject disjointness between test and train/validation sets; window-level outputs are aggregated to the session level, and ROC-based thresholding yields session-level deception/truth decisions.

Figure 1 provides an overview of the end-to-end workflow used in this study. All analyses start from the LieWaves dataset, which provides two approximately 75 s sessions (truth and deception) per participant recorded with a five-channel head-mounted EEG device. Raw CSV files are first passed through a common preprocessing pipeline that applies 50 Hz notch filtering, 1–45 Hz band pass filtering, and automatic wavelet-packet artifact reduction (ATAR), followed by basic consistency checks. Because the signals are subsequently band-limited to 1–45 Hz, the 50 Hz notch is effectively redundant under this setting; it is retained primarily for consistency with common EEG preprocessing conventions and prior LieWaves-related preprocessing descriptions, and to mitigate any residual line-noise leakage due to finite filter roll-off. Under a 1–45 Hz pass-band, omitting the 50 Hz notch is not expected to materially affect the downstream features or performance. No re-referencing, ICA decomposition, or manual artifact rejection is introduced at this stage.

From the ATAR-preprocessed continuous recordings, we derive several windowed representations that support both descriptive analyses and the two main modeling regimes. For descriptive statistics and classical cross-subject baselines, we extract 2.0 s windows (1.0 s hop) and compute band-power and time-domain descriptors (Section 2.4). For the subject-dependent OSW experiments, we segmented each session into 2.0 s windows (0.25 s hop), z-scored each window per channel, and computed DWT features and an extended spectral–statistical FEATS vector in parallel with the raw windows. For the subject-independent deep and spatial-filter pipelines, we built two additional window sets from the same preprocessed recordings: 3.0 s windows (0.25 s hop) for the grouped cross-validation ResNet-SE model, and 4.0 s windows (0.5 s hop) for the LOSO-based CNN and spatial-filter baselines (Section 2.3).

Feature extraction then proceeds along two levels of complexity. At the descriptive level, we computed per-window band powers in the canonical δ, θ, α, β, and γ ranges, Hjorth parameters, simple band ratios, and cross-channel aggregates, yielding a 65-dimensional interpretable feature space used for exploratory statistics and classical cross-subject models. At the OSW level, we computed richer 4-level db4 DWT descriptors (175 dimensions) and an extended 167-dimensional FEATS vector that includes finer-grained bands, relative powers, spectral entropy, Hjorth parameters, differential entropies, and frontal asymmetry indices (Section 2.4). These hand-crafted features are used both directly by classical models and as auxiliary inputs to the hybrid deep architecture in the subject-dependent setting.

Modeling and evaluation are organized into two complementary branches. In the subject-dependent OSW branch, sessions are pooled across subjects and partitioned once using a stratified 80/10/10 split over recordings (sessions). All windows extracted from a session inherit its subset assignment, while sessions from the same subject may still appear across subsets. The proposed Res-TCN-SE-Attention model operates on RAW + DWT + FEATS inputs and is compared against several CNN baselines trained on raw windows and classical models trained on FEATS only (Section 2.5.1 and Section 2.5.3). Performance is assessed primarily at the window level, with additional session-level aggregation of window probabilities for the best OSW model.

In the subject-independent branch, we enforce disjoint subject sets between the test fold and the training/validation pool in each split. The main ResNet-SE model is trained under grouped five-fold cross-validation by subject on 3.0 s RAW windows, with light data augmentation and focal loss; window-level probabilities are aggregated to the session level and converted to labels using a global ROC-based threshold derived from cross-validated scores. Additional CNN and spatial-filter baselines (ShallowConvNet, InceptionTime-SE, EEGNet++, FBCSP + LDA, and Riemann TS + LR) operate on 4.0 s RAW windows under a leave-one-subject-out (LOSO) protocol, and classical models are trained on session-level averages of the 2.0 s descriptive feature vectors. In this branch, session-level accuracy is treated as the primary outcome, as it reflects the operational task of deciding whether a given recording corresponds to deception or truth.

### 2.3. Signal Preprocessing and Segmentation

All recordings were acquired with a five-channel head-mounted device (AF3, T7, Pz, T8, and AF4; sampling rate of 128 Hz). To attenuate device and environment artifacts while keeping the pipeline simple and reproducible, we applied a two-stage offline filtering chain to every session. First, a 50 Hz infinite impulse response (IIR) notch filter with quality factor Q=30 was used to suppress mains interference. Second, the signal was band-limited to 1–45 Hz using a 4th-order Butterworth band pass filter. Both filters were implemented with zero-phase forward–reverse filtering (SciPy filtfilt), which removes phase delay by filtering once in the forward direction and once on the time-reversed signal, effectively squaring the magnitude response (resulting in a 4th-order notch and an 8th-order band pass). Reflection padding was used to reduce edge artifacts.

Third, we applied an ATAR step to the filtered signals, independently for each EEG channel. For ATAR, each channel was decomposed by wavelet-packet analysis using a Daubechies-4 (db4) mother wavelet up to the maximum level permitted by the signal length. At the deepest decomposition level, we obtained a set of frequency-ordered terminal nodes (wavelet packets). For each packet, we computed a relative soft-threshold equal to a fixed fraction (10%) of the maximum absolute coefficient in that packet. Coefficients with magnitude below this threshold were shrunk toward zero, while larger coefficients were reduced in magnitude but preserved in sign. The denoised channel signal was then reconstructed from the thresholded wavelet packets, with minor length mismatches corrected by trimming or edge padding where necessary. This procedure aims to attenuate high-amplitude, transient artifacts while preserving the overall spectral content within the 1–45 Hz band and is applied consistently across all sessions using fixed parameters (db4 wavelet, relative threshold 0.1). No re-referencing, Independent Component Analysis (ICA) or manual artifact rejection was performed.

**Figure 2:**
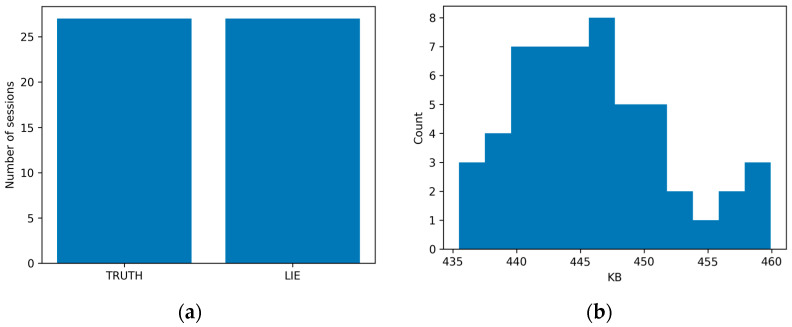
Basic dataset diagnostics for LieWaves: (**a**) session-level class distribution (27 truth vs. 27 lie sessions); and (**b**) histogram of raw EEG file sizes (kB) across all 54 sessions, indicating broadly consistent recording duration.

Basic book-keeping confirmed 27 unique participants and 54 sessions with balanced truth/lie labels, and broadly consistent recording lengths across sessions (Figure 2). All subsequent analyses (descriptive statistics, classical models, and deep-learning pipelines) operated on these ATAR-preprocessed, band-limited signals.

After preprocessing, each continuous session was segmented into fixed-length windows using a sliding window. A window was retained only if it contained the full duration (partial segments at the recording end were discarded). In all deep-learning and short-window feature analyses, each window was standardized per channel to a zero mean and unit variance before feature extraction or modeling. For descriptive band power features used in univariate analyses and session-level classical baselines, features were computed directly on the ATAR-preprocessed, band-limited signals without additional per-window z-scoring.

For the subject-dependent OSW experiments, we used 2.0 s windows with a 0.25 s hop (256 samples per window). For the subject-independent ResNet-SE models, we likewise operated on overlapping fixed-length windows extracted from the ATAR-preprocessed sessions but used a 3.0 s temporal context, corresponding to 3.0 s windows (384 samples) with a 0.25 s hop. Additional cross-subject convolutional and classical baselines (Section 2.5.3) explored a longer 4.0 s context with a 0.5 s hop. In all cases, labels are defined at the session level in the original dataset, and each window inherits the label of its parent session. For cross-subject experiments, window-level outputs are aggregated back to session-level scores by averaging the predicted probabilities across all windows belonging to a session; decision thresholds for converting these scores to binary session-level predictions are derived from cross-validated session statistics as described in Section 2.6.3.

**Figure 3:**
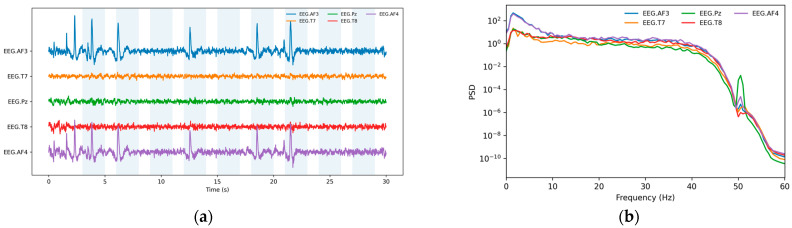
Subject 1, session 1: (**a**) 30 s excerpt of an automatic wavelet-packet artifact reduction (ATAR)-preprocessed recording on the five EEG channels (shaded bars mark stimulus-on intervals); and (**b**) PSD for the same session and channels, showing out-of-band attenuation consistent with the 1–45 Hz pass-band (the additional 50 Hz notch is redundant under this range), as well as between-channel spectral differences (log-scale *y*-axis).

The overall effect of the preprocessing chain is illustrated in Figure 3, which shows a 30 s excerpt of an ATAR-preprocessed recording together with the corresponding power spectral densities (PSDs) for the same session and channels. As expected, out-of-band activity is attenuated primarily by the 1–45 Hz band pass (the additional 50 Hz notch is redundant under this range), while ATAR visibly reduces isolated high-amplitude transients and preserves the canonical EEG spectral structure across channels.

Filter, ATAR, and window parameters were chosen to balance simplicity, preservation of relevant EEG content, and deployment realism rather than to exhaustively optimize performance. The 1–45 Hz band pass is a common choice in cognitive and BCI-oriented EEG [66], retaining delta through low-gamma activity while attenuating slow drifts and higher-frequency muscle-related components. A narrow 50 Hz notch was retained for consistency with common preprocessing templates and to mitigate any residual line-noise leakage due to finite filter roll-off, although with a 45 Hz upper cutoff, it has negligible practical impact in this study. The wavelet-packet soft-thresholding step provides a generic, fully automatic artifact attenuation without introducing device- or subject-specific tuning. The final configurations (2.0 s/0.25 s in the subject-dependent OSW setting, 3.0 s/0.25 s for the main subject-independent ResNet-SE model, and 4.0 s/0.5 s for some additional baselines) reflect a practical trade-off between retaining sufficient low-frequency content, generating enough training segments, and limiting computational cost. The 0.25 s and 0.5 s hops increase the number of training samples at the cost of higher correlation between adjacent windows; this trade-off is intentional in the subject-dependent OSW setting and is explicitly acknowledged when interpreting window-level performance.

### 2.4. Feature Extraction and Descriptive Analysis

In addition to end-to-end learning on raw, z-scored windows, we computed a compact set of interpretable descriptors tailored to low-channel wearable recordings. All features were computed on the ATAR-preprocessed, band-limited EEG described in Section 2.3.

For descriptive analyses and some classical baselines, we extracted band-power and time-domain descriptors on fixed 2.0 s windows with a 1.0 s hop (256 samples and 128-sample step at 128 Hz). For each such window and channel, we estimated the PSD using Welch’s method (SciPy implementation) with a Hann window, constant detrending, and segment length capped at 256 samples (min (256,N)). Thus, a 2.0 s window at 128 Hz uses a single Hann segment, whereas longer segments (when present) are decomposed into a small number of overlapping segments, which stabilizes the PSD estimate without introducing many additional hyperparameters. Band powers were obtained by integrating the PSD over canonical ranges, 1–4 Hz (delta, δ), 4–8 Hz (theta, θ), 8–13 Hz (alpha, α), 13–30 Hz (beta, β), and 30–45 Hz (gamma, γ) [67,68], using the trapezoidal rule [69]. From the time domain, we extracted Hjorth activity, mobility, and complexity for each channel, and we formed simple spectral ratios such as β/α and θ/α that are commonly used in vigilance and control-demand paradigms.

**Figure 4:**
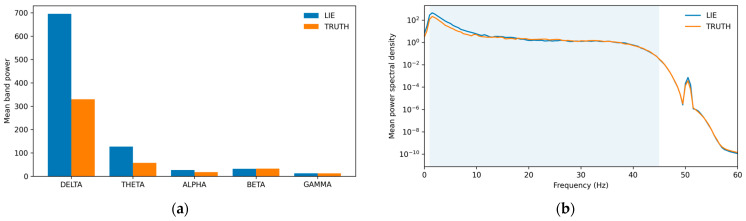
Subject 1, session 1: (**a**) mean band power over 2.0 s automatic wavelet-packet artifact reduction (ATAR)-preprocessed windows, averaged across the five EEG channels for lie and truth segments; and (**b**) mean PSD across the same channels for the same session, with the 1–45 Hz pass-band highlighted (log-scale *y*-axis).

To summarize patterns in a headset with limited spatial sampling, we also computed channel-aggregated statistics by taking the mean and standard deviation (std) of each band across the five electrodes in every window. These aggregates capture coarse cross-channel structure while being relatively robust to occasional local artifacts. For illustration, Figure 4 shows, for one representative subject, cross-channel mean band powers and mean PSD by class: differences between lie and truth segments are most apparent at lower frequencies, while the overall spectra remain closely overlapping. Overall, these descriptive features show substantial overlap between conditions and are therefore used mainly as an interpretable baseline and for classical models, rather than as evidence of strong standalone separability. The resulting feature matrix has 65 columns per window (per-channel band powers, Hjorth parameters, band ratios, and cross-channel aggregates, plus metadata), and is used both to characterize class-wise trends (Figure 4, Table 2 and Table 3) and as an interpretable feature space for cross-subject classical baselines (logistic regression (LR), support vector machine (SVM), random forest (RF) and related classical classifiers) under grouped cross-validation by subject.

For the short-window, subject-dependent OSW experiments, we enriched the raw-signal input with two complementary hand-crafted feature vectors. First, for each 2.0 s segment and channel, we computed a 4-level DWT with a Daubechies-4 (db4) mother wavelet (PyWavelets). This yields one approximation and four detail sub-bands that together provide a compact multiresolution view of the signal at 128 Hz. From each sub-band, we extracted six summary statistics (minimum, maximum, median, mean, standard deviation, and variance) and the relative energy (sub-band energy divided by the total across bands), giving 35 values per channel and 175 values across the five electrodes.

Second, we constructed a classical spectral–statistical feature vector, hereafter denoted FEATS, using extended frequency bands. For each channel, band powers were computed with Welch’s method over the bands δ, θ, α, $\alpha_{low}$, $\alpha_{high}$, β, $\beta_{low}$, $\beta_{high}$, γ, $\gamma_{low}$, and $\gamma_{middle}$, covering 0.5–65 Hz overall. Given the 1–45 Hz band pass preprocessing, contributions above ≈45 Hz are strongly attenuated and mainly capture residual energy near the upper edge of the pass-band. From these powers, we derived relative band powers (normalized by total power), θ/β and (θ+α)/β ratios, spectral entropy, Hjorth activity, mobility and complexity, and band-limited differential entropies for δ, θ, α, β and γ. Finally, we added two frontal asymmetry indices based on log α and β power differences between AF4 and AF3. The resulting FEATS vector contains 167 values per window (33 descriptors per channel plus two asymmetry terms), providing a compact summary of extended-band spectral content and simple temporal statistics on OSWs. These descriptors are used as inputs for the classical baselines and as auxiliary inputs in the hybrid model described in Section 2.5 and Section 2.6.

Although these handcrafted descriptors provide an interpretable baseline, they compress each window into coarse summary statistics and therefore discard within-window temporal structure (e.g., transient motifs, waveform morphology, and phase-dependent patterns) as well as nonlinear cross-channel interactions. By contrast, the deep models in Section 2.5 operate directly on the raw time series (and complementary multiscale summaries), allowing them to learn discriminative temporal patterns end-to-end. This helps reconcile the limited separability seen in the descriptive feature space with the strong subject-dependent performance observed for end-to-end models under OSW evaluation.

To quantify global differences between truth and lie, we pooled the 2.0 s band-power windows across channels and sessions and examined the distribution of cross-channel mean band powers. Table 2 reports quartiles and interquartile ranges (IQRs) for each band and class. IQRs are the largest in the δ and θ bands, indicating substantial between-subject variability. Median class differences are small relative to these IQRs (e.g., modest shifts in beta and gamma power toward TRUTH and in δ and θ toward LIE), and the corresponding Cliff’s Δ effect sizes remain |Δ|≤0.088 across all bands (Table 3). Taken together, these summary statistics indicate that pooled univariate band power alone offers only weak separability at the level of descriptive statistics, which is consistent with the moderate cross-subject generalization performance observed later.

The specific parameter choices in the feature-extraction pipeline were guided by pragmatic considerations of stability, simplicity, and deployment realism rather than exhaustive optimization. Welch’s method was chosen because it provides low-variance PSD estimates with a small number of intuitive parameters and is standard in EEG analysis. Capping the segment length at 256 samples gives a frequency resolution of 0.5 Hz at 128 Hz sampling, sufficient for integrating over the canonical bands while keeping computations inexpensive. The Hann window and simple detrending avoid edge artifacts without strong assumptions about the underlying signal, and the coarse band integrals are relatively insensitive to the exact choice of window and overlap. The 4-level db4 DWT provides a compact, multiscale description on 2.0 s windows, with good time–frequency localization and a long history in EEG feature extraction. Using compact feature summaries and lightweight transforms helps limit capacity and overfitting risk while retaining complementary information to the raw signal.

### 2.5. Model Architectures

We considered three main families of models: (i) a temporal convolutional network with residual, squeeze-and-excitation (SE), and attention mechanisms for the subject-dependent overlapping sliding-window setting; (ii) a compact residual unidimensional convolutional neural network (1D-CNN) with SE for the subject-independent setting; and (iii) a set of standard CNN and classical machine-learning baselines operating on raw windows or on hand-crafted feature vectors. All models take as input fixed-length windows extracted from the preprocessed EEG, with windowing schemes defined in Section 2.3 and training/evaluation protocols in Section 2.6. Deep models in both branches operate on ATAR-denoised signals, while some classical cross-subject baselines use features computed directly from band-pass + notch-filtered recordings.

#### 2.5.1. Res-TCN-SE-Attention Subject-Dependent Model

For the subject-dependent (session-disjoint) OSW experiments, we used a residual temporal convolutional network with depthwise-separable convolutions, squeeze-and-excitation blocks, and attention pooling. The Res-TCN-SE-Attention model receives a 2.0 s window of ATAR-preprocessed, z-scored EEG of shape T×C (256 samples at 128 Hz, 5 channels) and processes it through a raw branch and two feature branches (DWT and FEATS).

In the raw branch, the input is first perturbed with low-variance Gaussian noise (std 0.01 in z-score units) and passed through an initial Conv1D layer (64 filters, kernel size 5, and “same” padding). This is followed by a stack of five depthwise-separable residual blocks. Each block applies two SeparableConv1D layers with batch normalization, ReLU activation, and spatial dropout, wrapped in a residual connection that includes a 1 × 1 convolution whenever the number of filters changes. An SE block modulates each block’s output by global average pooling across time, passing the pooled vector through a small two-layer gating MLP (bottleneck size max(f/8, 8)) and rescaling the feature maps by the resulting channel-wise weights. Across the five blocks, the number of filters and dilation factors increase (64, 96, 128, 192, and 256 filters with dilations 1, 2, 4, 8, and 12), and the first four blocks apply MaxPool1D (factor 2) to progressively downsample the time axis, while the last block leaves the temporal resolution unchanged.

After the final residual block, an attention-pooling module aggregates temporal information. A 1 × 1 Conv1D with tanh activation produces an attention score per time step, followed by a second 1 × 1 convolution and a Softmax over time. These attention weights are used to compute a weighted sum of the feature maps via element-wise multiplication and summation over time, yielding a fixed-size context vector. A dense layer with 192 ReLU units, with dropout applied before and after, produces the raw-branch embedding.

In parallel, the DWT branch receives the 175-dimensional vector described in Section 2.4 (4-level db4 DWT statistics and relative energies across five channels). This vector is normalized (LayerNormalization) and passed through a small two-layer MLP (Dense 256 → Dropout → Dense 128 with ReLU activations), producing a compressed DWT embedding.

The FEATS branch operates analogously on the 167-dimensional FEATS vector (extended-band spectral–statistical features and frontal asymmetries), using LayerNormalization, a dense layer with 192 units, dropout, and a second dense layer with 128 units (all ReLU).

**Figure A1:**
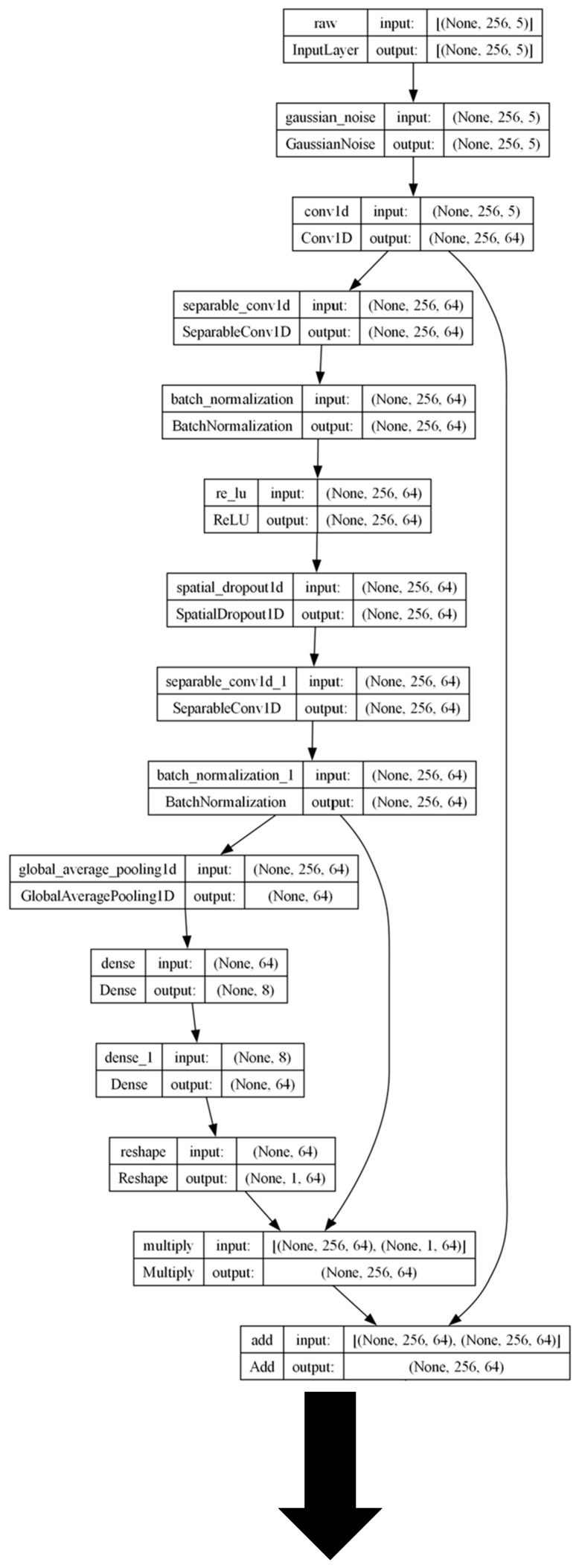

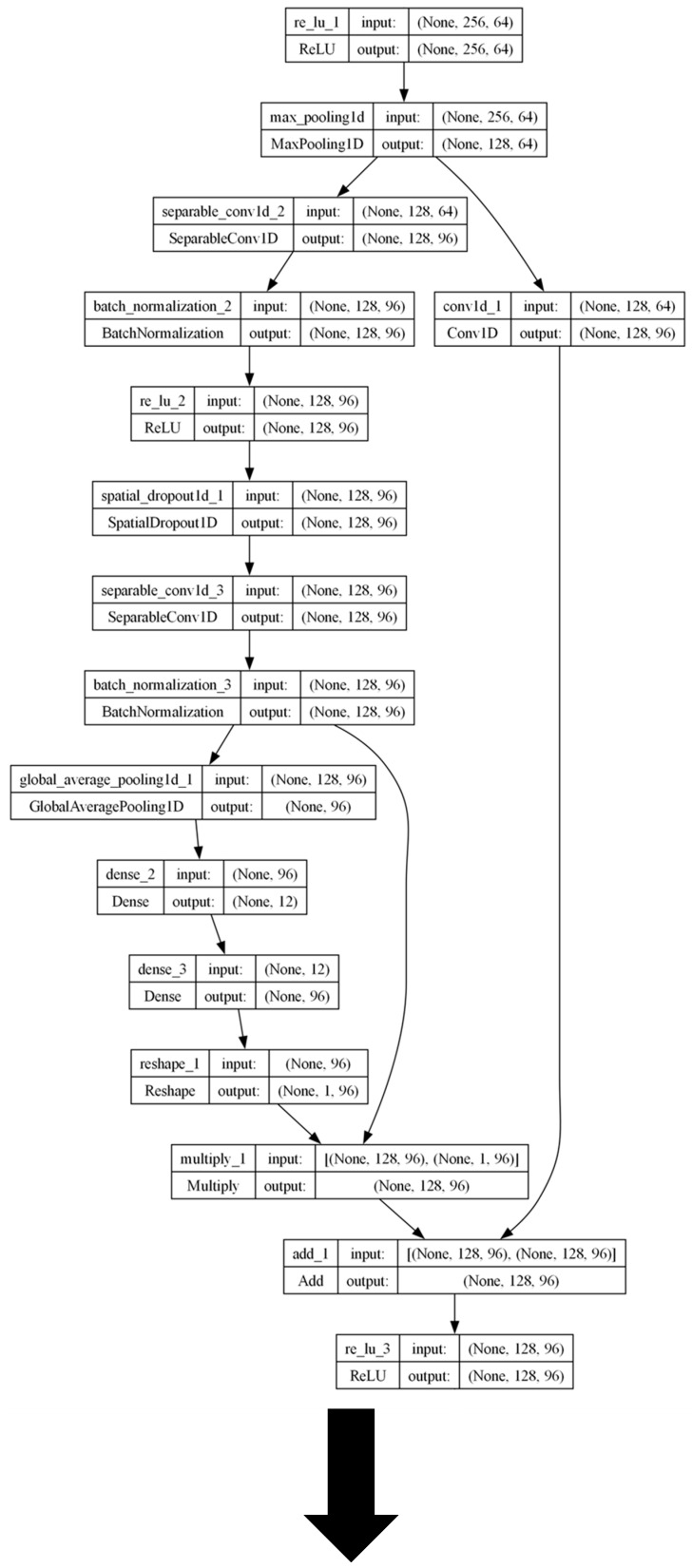

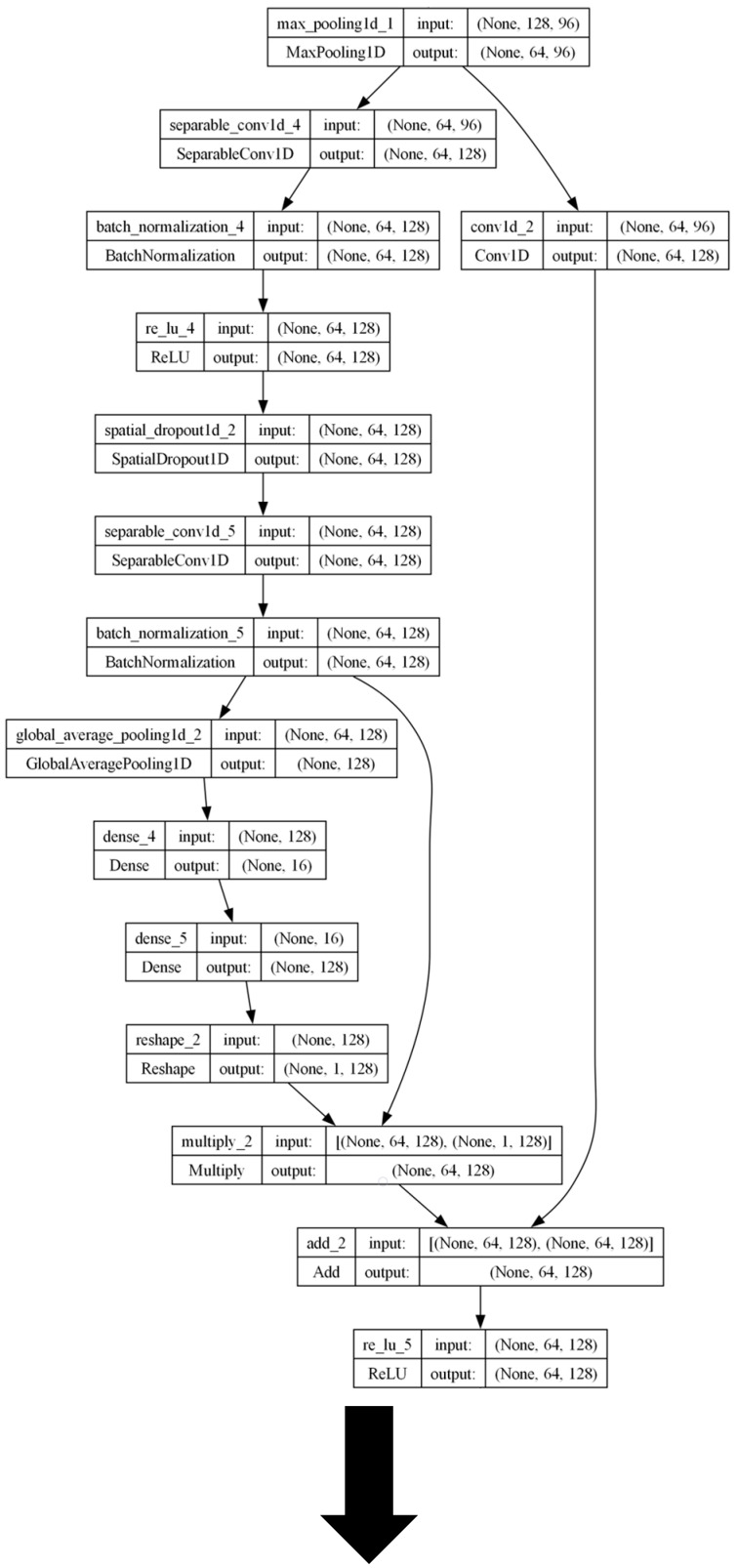

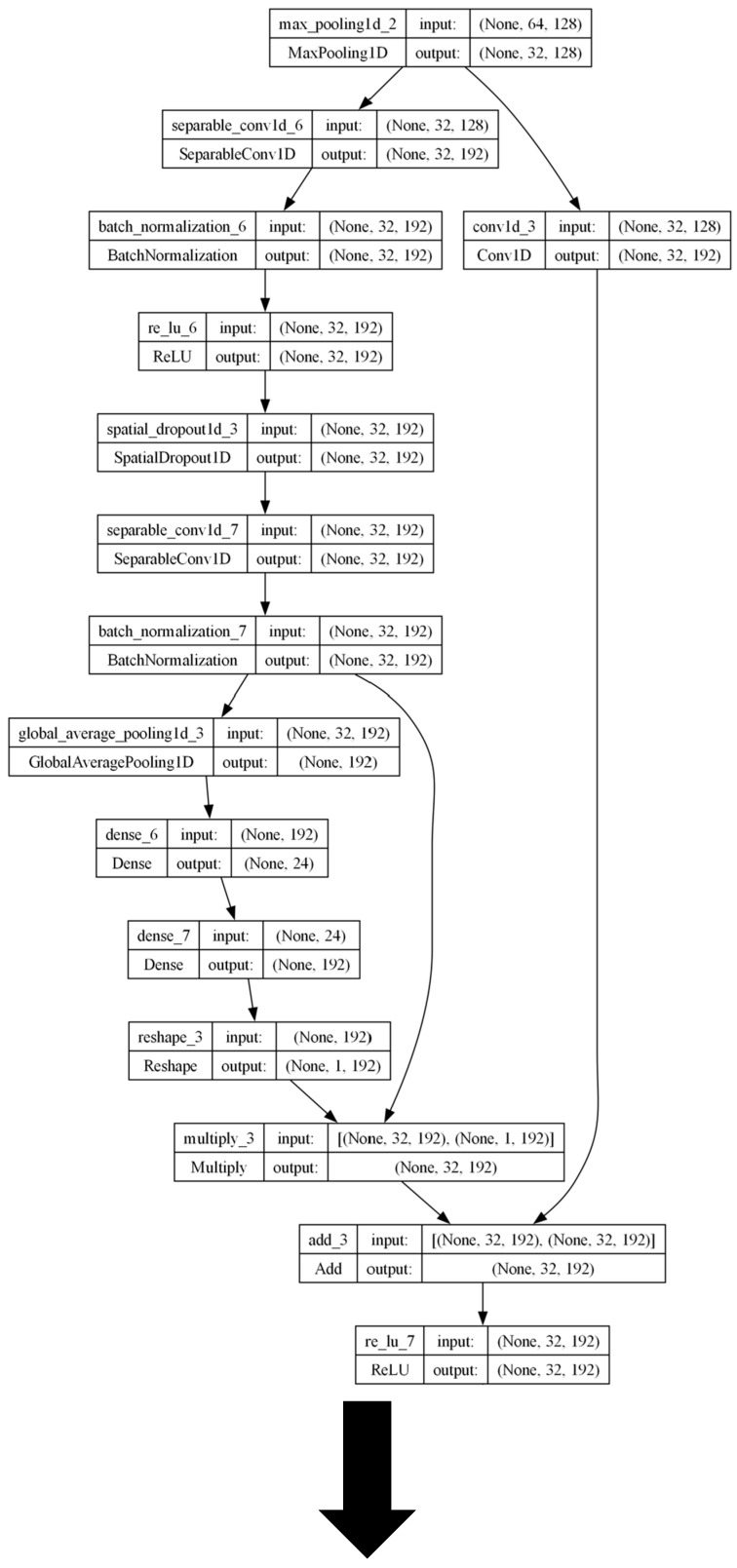

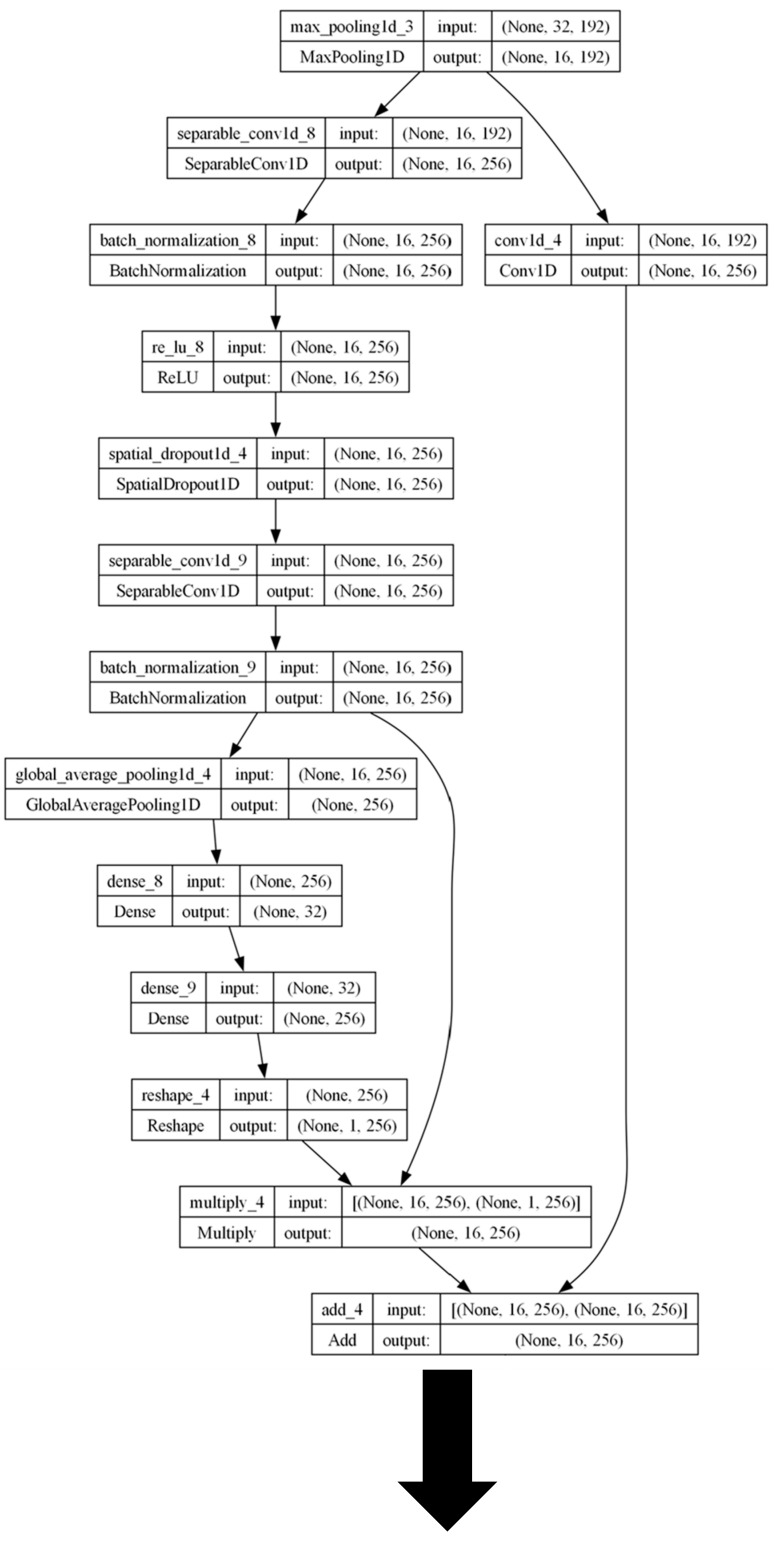

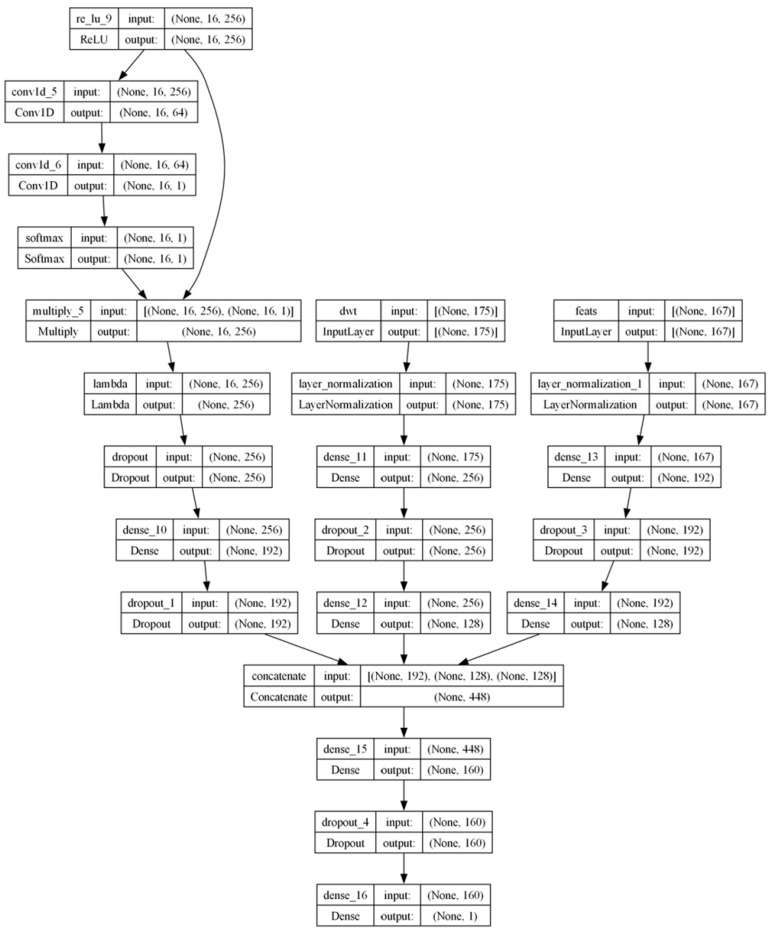
Architecture of the Residual Temporal Convolutional Network with Squeeze-and-Excitation and Attention (Res-TCN-SE-Attention) model for deception detection from automatic wavelet-packet artifact reduction (ATAR)-preprocessed EEG. The raw waveform branch (top) receives 2.0 s windows (256 samples × 5 channels at 128 Hz) and passes them through Gaussian noise injection, an initial Conv1D layer, and five depthwise-separable residual blocks with squeeze-and-excitation (SE) modulation and progressive temporal down-sampling, followed by attention pooling and a 192-unit dense layer. In parallel, a DWT branch encodes a 175-dimensional wavelet feature vector with a small multi-layer perceptron, and a FEATS branch encodes a 167-dimensional extended spectral–statistical feature vector. The three embeddings are concatenated and passed through a 160-unit dense layer with dropout and a final sigmoid neuron that outputs the deception probability for each window.

The three embeddings (raw, DWT, and FEATS) are concatenated and fed to a classification head consisting of a dense layer with 160 ReLU units, dropout, and a final 1-unit sigmoid output. This design keeps the handcrafted representations low-dimensional while allowing the network to learn how much weight to assign to wavelet- and FEATS-based information relative to the raw signal. The full architecture of the Res-TCN-SE-Attention model is illustrated in Figure A1. This architecture keeps the model relatively compact while allowing it to leverage complementary information from raw waveforms, multiscale wavelet features, and extended-band spectral–statistical descriptors in the subject-dependent OSW setting. Training details (loss, optimizer, learning-rate schedule, and subject-dependent splits) are given in Section 2.6.2.

#### 2.5.2. ResNet-SE Subject-Independent Model

For the subject-independent experiments, we used a compact residual 1D-CNN with squeeze-and-excitation. The ResNet-SE network operates on fixed-length windows of ATAR-preprocessed EEG of shape T×C; in the main configuration, we used 3.0 s windows at 128 Hz (384 samples × 5 channels), extracted with a 0.25 s hop (Section 2.3). Each window is z-scored per channel before being fed to the network. The goal was to balance representational capacity with a footprint suitable for potential deployment on resource-constrained hardware.

**Figure A2:**
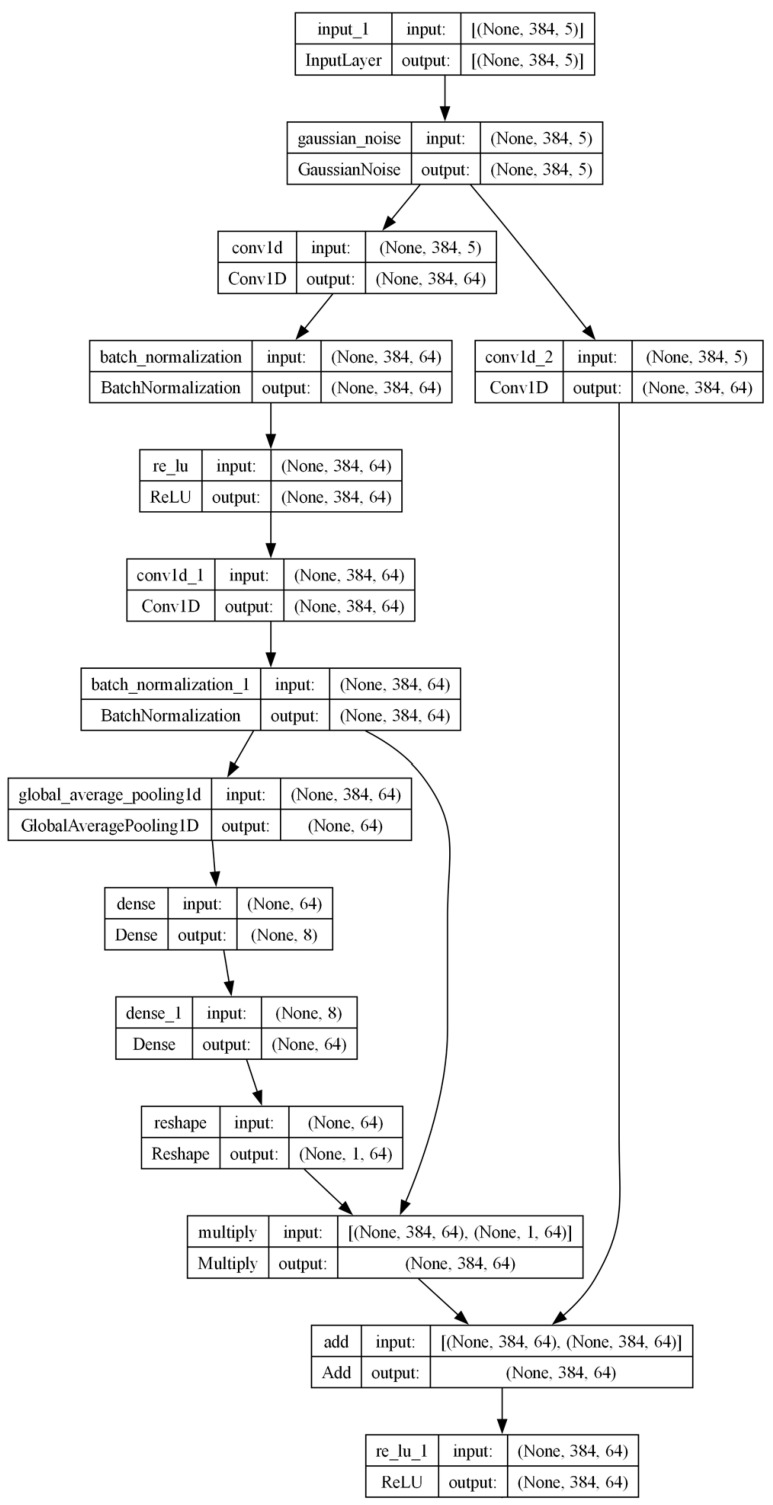

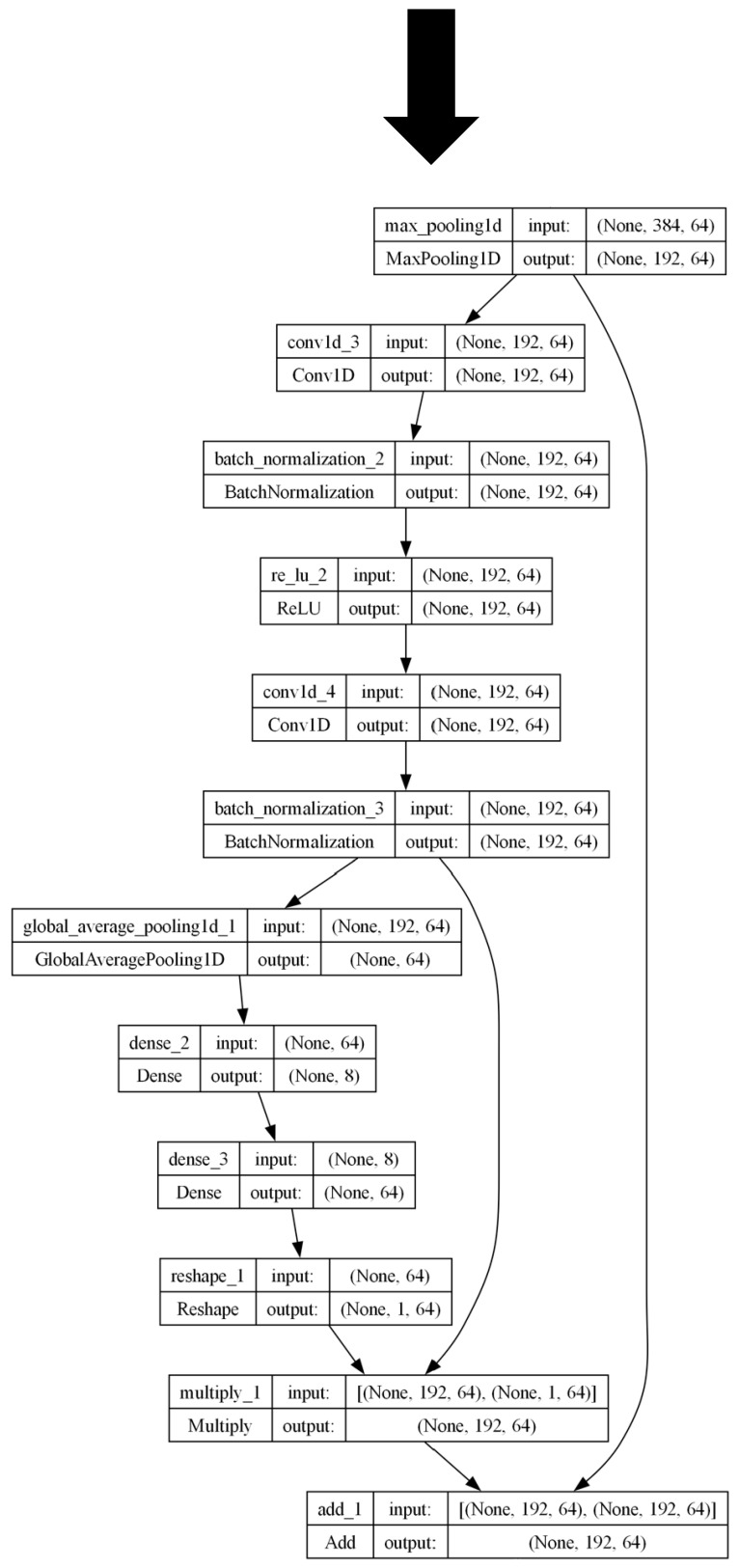

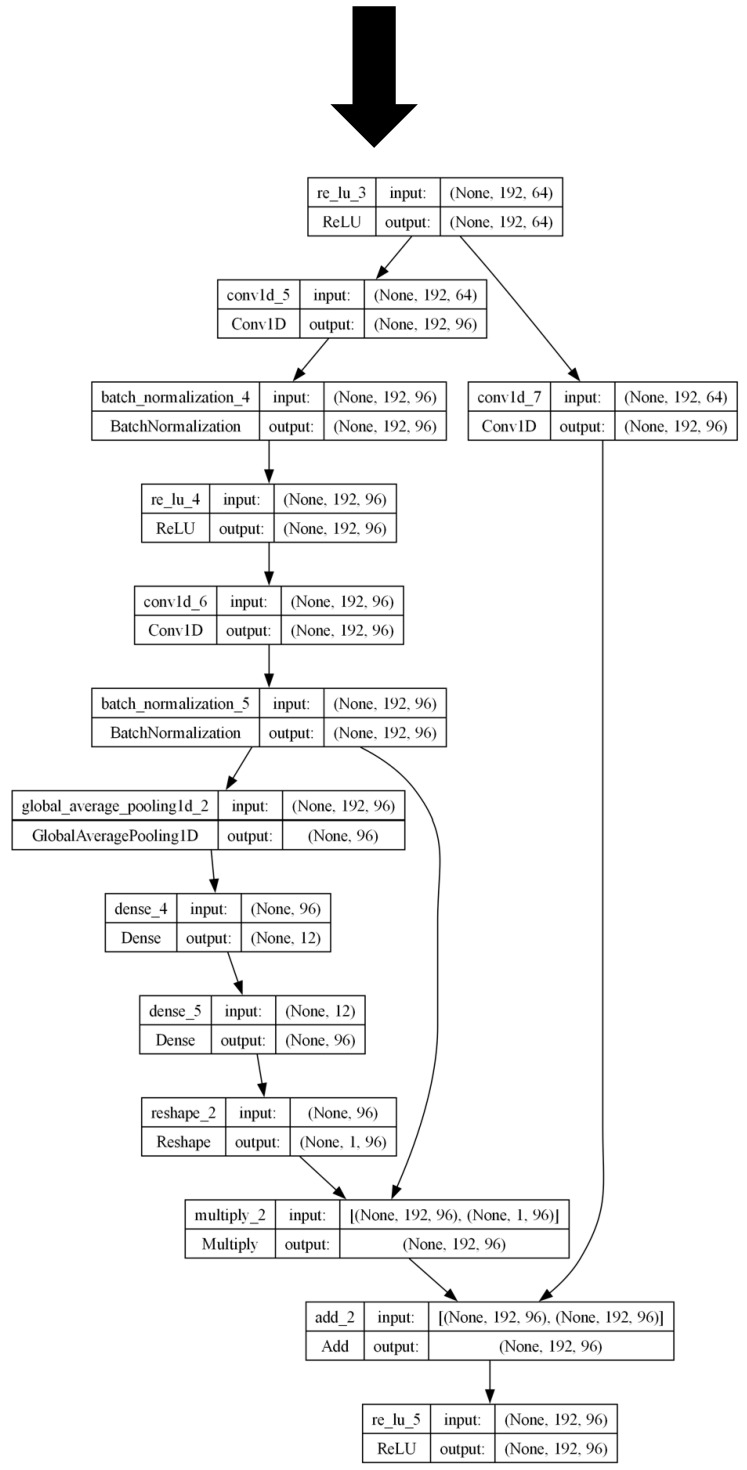

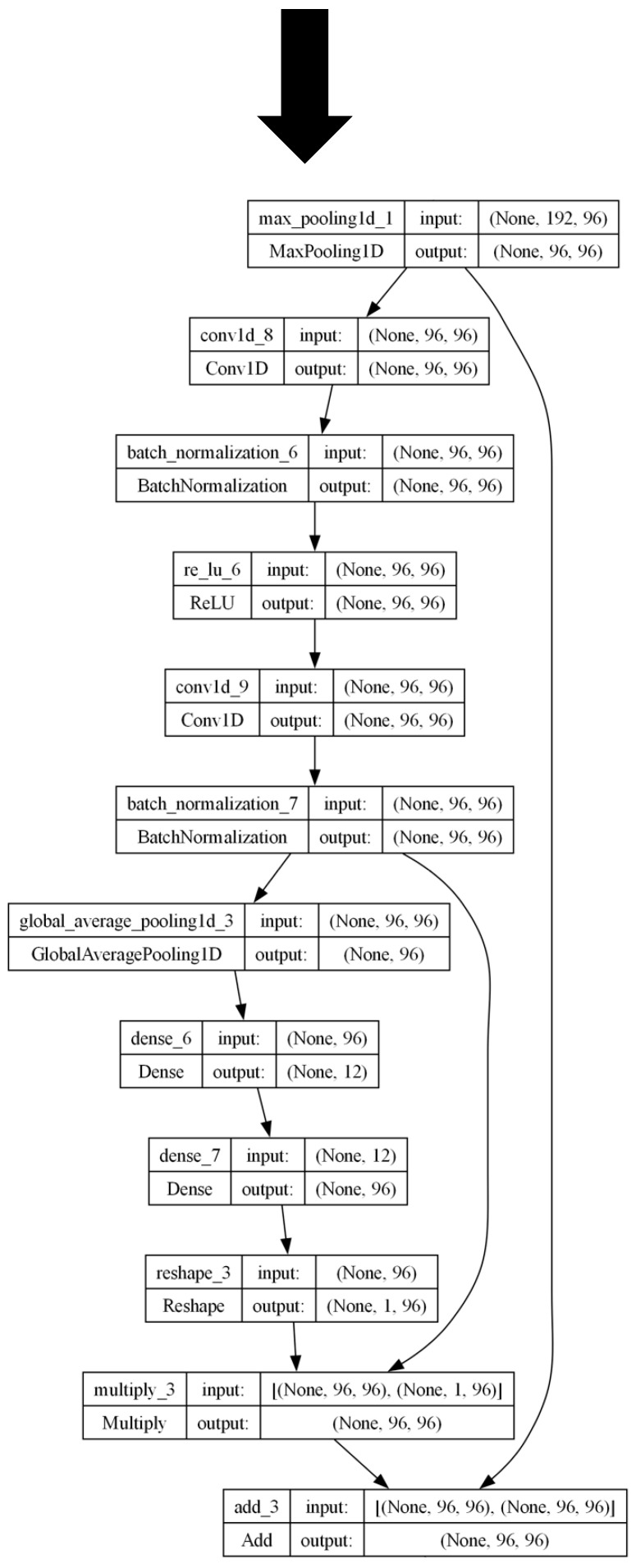

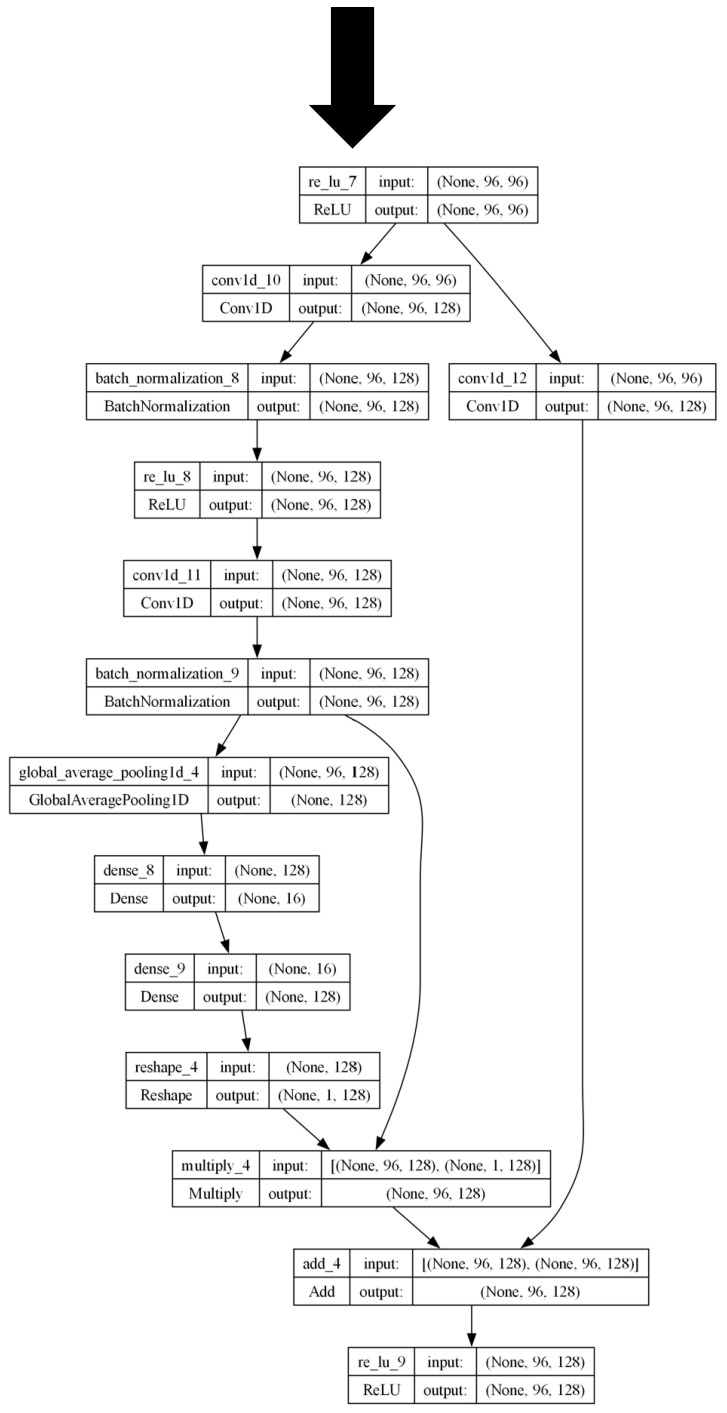

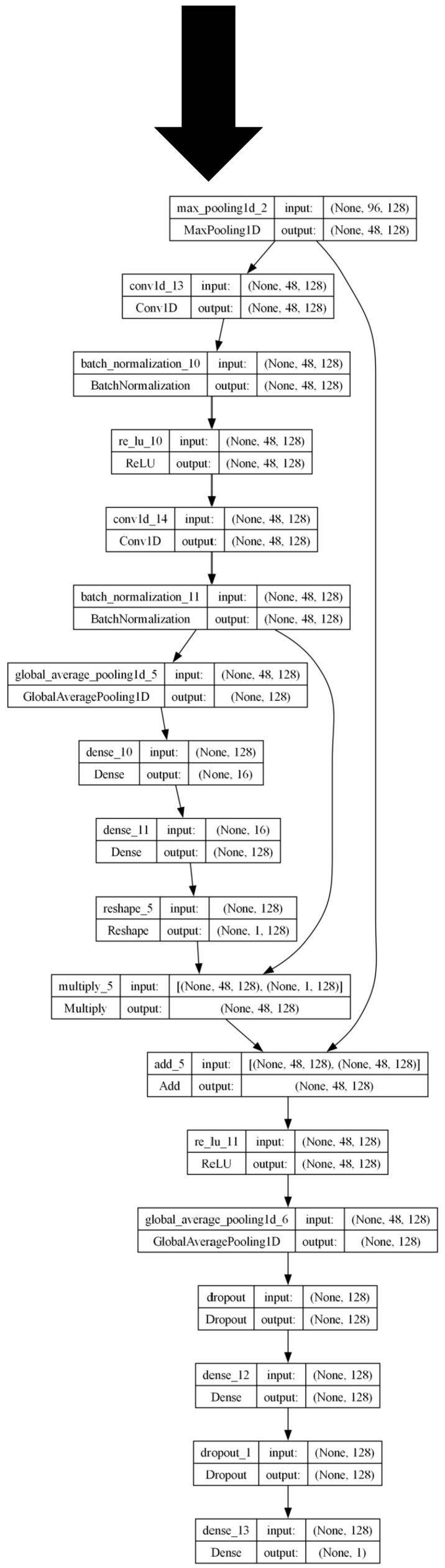
Architecture of the Residual Network with Squeeze-and-Excitation blocks (ResNet-SE) model. The network is a three-stage residual 1D-CNN with squeeze-and-excitation (SE) blocks operating on automatic wavelet-packet artifact reduction (ATAR)-preprocessed 3.0 s EEG windows (384 samples × 5 channels at 128 Hz). Each stage comprises two dilated Conv1D residual blocks (64, 96, and 128 filters with kernel sizes 11, 9, and 7, respectively), with temporal down-sampling after the first block in each stage. The final feature maps are aggregated with global average pooling, followed by dropout, a 128-unit dense layer, and a 1-unit sigmoid output for deception versus truth classification.

The input is first passed through a Gaussian noise layer (std 0.01 in z-score units) and then fed to three convolutional stages, each composed of two residual blocks with increasing width and decreasing kernel sizes. In stage 1, the blocks use 64 filters with kernel size 11 and dilation 1; in stage 2, they use 96 filters with kernel size 9 and dilation 2; and in stage 3, they use 128 filters with kernel size 7 and dilation 4. Within each residual block, two Conv1D layers are applied in sequence, each followed by batch normalization and ReLU activation. An SE block modulates the block output via global average pooling, a two-layer gating MLP with bottleneck size max(f/8, 8), and channel-wise multiplication. If the number of channels differs between input and output, the shortcut path is projected with a 1 × 1 Conv1D so that the residual addition is well-defined. In each stage, the first block includes a MaxPool1D layer (factor 2) at the end, reducing temporal resolution and expanding the effective receptive field, while the second block leaves the current temporal resolution unchanged. The full architecture of the ResNet-SE model is illustrated in Figure A2.

After the last residual block, a GlobalAveragePooling1D layer aggregates features across time, followed by dropout and a dense layer with 128 ReLU units. A final dropout layer and a 1-unit sigmoid output complete the classifier. The model is trained with the Adam optimizer and a binary focal loss (α = 0.25, γ = 2.0) to down-weight well-classified windows and focus on harder examples. During training, a data pipeline applies shuffling, light Gaussian noise injection, and mixup augmentation to training batches, while a ReduceLROnPlateau scheduler and model checkpointing track the validation area under the ROC curve (AUC) for learning-rate adaptation and model selection.

Subject-wise grouped K-fold cross-validation is used to evaluate generalization to unseen participants. In each fold, a subset of subjects is held out entirely as the test set, and the remaining subjects provide training and validation windows; all windows from a given subject appear either in the test set or in the training/validation pool of that fold, never in both. Within the training/validation pool, a small fraction of windows (≈10%) is reserved for validation at the window level. After training, window-level scores from the best epoch are aggregated to the session level by averaging the predicted probabilities over all windows belonging to each session. A single global decision threshold is then estimated from the cross-validated session-level scores across folds using ROC-based criteria, and this threshold is applied to the aggregated session probabilities to obtain subject-independent deception/truth decisions (Section 2.6.3).

#### 2.5.3. Additional CNN and Classical Baselines

To contextualize the performance of the two main architectures, we included additional deep and classical baselines in both subject-dependent and subject-independent settings.

For the subject-dependent OSW experiments (Table 4), we trained several standard CNN architectures on raw 2.0 s windows with a 0.25 s hop, using the same ATAR-preprocessed inputs as the Res-TCN-SE-Attention model: a shallow 1D-CNN, a one-dimensional residual network (ResNet1D), an InceptionTime-style 1D convolutional network (InceptionTime1D), a lightweight deep convolutional network (DeepConvNet-lite), and an EEG-specific convolutional neural network (EEGNet). These implementations follow their published designs, adapted to five-channel, 128 Hz EEG (Conv1D layers with “same” padding, batch normalization, non-linearities and pooling, followed by a dense classifier head), trained on the same fixed 80/10/10 stratified session-level partition as the Res-TCN-SE-Attention model (all windows from a session inherit its subset assignment). In addition, classical baselines, LR, RF, k-nearest neighbors (k-NN), SVM with radial basis function kernel (RBF), gradient boosting decision trees (GB), and a simple MLP, operate solely on the 167-dimensional FEATS vector (Section 2.4), after standardization on the training set and using a validation-derived threshold for window- and session-level evaluation.

For the cross-subject setting (Table 5), we considered additional CNNs on raw windows using a LOSO protocol and longer 4.0 s windows with a 0.5 s hop: a Shallow Convolutional Network (ShallowConvNet), an InceptionTime network with squeeze-and-excitation blocks (InceptionTime-SE), and an enhanced EEGNet-style model with multi-band temporal kernels and attention (EEGNet++). These architectures are trained on ATAR-preprocessed, z-scored windows with light data augmentation (Gaussian noise, temporal scaling and shifts, channel dropout, and time masking). Validation ROC AUC is used for model selection, and where hard decisions are needed (e.g., for confusion matrices or accuracy), fold-specific cutpoints are derived from ROC analysis following the principles described for the main ResNet-SE model (Section 2.6.3).

In parallel, we implemented spatial-filtering pipelines: filter-bank common spatial patterns (FBCSP) followed by a shrinkage linear discriminant analysis (LDA) classifier, and Riemannian covariance features projected to the tangent space (TS) with a logistic-regression classifier. These methods operate on the same ATAR-preprocessed 4.0 s overlapping windows as the CNN baselines and are evaluated under the same LOSO protocol.

Finally, we evaluated a panel of classical cross-subject baselines on the 65-dimensional descriptive feature set derived from band powers, Hjorth parameters and simple ratios (Section 2.4), computed at the 2.0 s window level (1.0 s hop) with grouped cross-validation by subject. For these models, window-level feature vectors are first averaged within each recording to obtain a single 65-dimensional feature vector per session. The panel includes LR, SVM-RBF, a calibrated linear SVM, k-NN, linear and quadratic discriminant analysis (LDA, QDA), Gaussian Naive Bayes (GaussianNB), RF, extremely randomized trees (ExtraTrees), GB, and, where available, gradient-boosting variants including Extreme Gradient Boosting (XGBoost), Light Gradient Boosting Machine (LightGBM) and Categorical Boosting (CatBoost). For each model, session-level probabilities obtained from these averaged feature vectors are thresholded at a fixed cutpoint (0.5), providing a set of reproducible baselines against which the deep models can be compared. Training protocols and metrics for these baselines are summarized in Section 2.6.

### 2.6. Training and Evaluation Protocol

#### 2.6.1. Experimental Setup and Computing Environment

Experiments were implemented in Python 3.10.11 using TensorFlow/Keras 2.10.0 (DirectML backend), scikit-learn 1.7.2, NumPy 1.26.4, SciPy 1.15.3, pandas 2.3.3, and Matplotlib 3.10.7. Feature pipelines additionally used PyWavelets for the Daubechies-4 discrete wavelet transform and the gradient-boosting libraries LightGBM, XGBoost, and CatBoost for tree-based classifiers.

All runs were executed on a Lenovo ThinkPad P16 Gen 1 mobile workstation running Windows 11 Pro (version 25H2, build 26200.7171), equipped with a 12^th^ Gen Intel Core i9-12900HX CPU (16 cores, 24 threads), 32 GB DDR5 RAM, a 1 TB PCIe 4.0 NVMe SSD, and an NVIDIA RTX A4500 Laptop GPU with 16 GB GDDR6 memory. Deep models (all convolutional networks and the hybrid RAW + DWT + FEATS architecture) were trained on the GPU via the DirectML backend, while classical learners (linear, tree-based, and instance-based models) were trained on the CPU.

To support reproducibility, we used a fixed random seed (42) for all libraries that expose it, including Python’s pseudo-random generator, NumPy, and TensorFlow/DirectML. The same seed was used in all procedures that involve randomness, such as train/validation/test splitting, window shuffling, mini-batch generation, and stochastic data augmentation, which improves run-to-run consistency even though some low-level GPU operations may remain non-deterministic. Unless stated otherwise, this fixed seed was used for all main experiments; for the subject-dependent ablation study reported in Section 3.1, we additionally repeated training with three independent seeds (0–2) to quantify run-to-run variability under the same OSW split and hyperparameters. For each cross-subject fold, we recorded the mapping between subject IDs and the train/validation/test partitions and the validation-derived decision threshold based on Youden’s J statistic. We also stored the cross-validated session-level scores and the corresponding global threshold used for the aggregated subject-independent results.

To assess practical feasibility, we also quantified computational cost and model footprint. Specifically, we report trainable parameter count and serialized weight-file size (FP32), training cost (seconds per epoch and total wall-clock training time under the study’s standard 150-epoch training configuration), peak memory usage during training (process RAM and approximate device-level GPU memory as reported by the system monitor), and inference cost (milliseconds per window) for batch sizes 1 and 64. For the hybrid subject-dependent RAW + DWT + FEATS pipeline, we additionally report Python-level feature-extraction time and an end-to-end per-session runtime (preprocessing + feature extraction + inference + aggregation), as this more directly reflects operational use than model-only inference. The resulting computational-efficiency metrics are summarized in Section 3.3.

#### 2.6.2. Subject-Dependent Training and Evaluation

In the subject-dependent OSW setting, each LieWaves recording (session) was treated as a coherent unit for dataset partitioning. We first partitioned sessions into training/validation/test sets using a single stratified 80/10/10 split over session IDs (stratified by the session label), and assigned all extracted windows to the same subset as their parent session (i.e., no temporal segment from a given recording can appear in more than one subset, preventing overlapping windows from crossing the train–test boundary). ATAR-preprocessed, band-limited EEG was segmented into overlapping 2.0 s windows with a 0.25 s hop (87.50% sample overlap) at 128 Hz (256 samples per window), and each window was z-scored channel-wise. Windows inherited the binary lie/truth label of their parent session.

For comparability across subject-dependent models (deep and classical), we used one fixed session-level partition and reused it across all experiments. Because each participant contributes two sessions, sessions from the same participant may occur across subsets; accordingly, this protocol remains “subject-dependent” while keeping each recording self-contained within a single subset. A summary of the subject-dependent models and configurations evaluated under this session-disjoint OSW is provided in Table 4.

We considered three input regimes: (1) RAW—end-to-end learning directly from the z-scored raw windows (2.0 s, 0.25 s hop), without additional handcrafted features; (2) FEATS—the 167-dimensional spectral–statistical feature vector defined in Section 2.4 (extended band powers, relative powers, power ratios, spectral entropy, Hjorth parameters, differential entropies, and frontal asymmetry indices), standardized with a scaler fitted on the training windows; and (3) RAW + DWT + FEATS—the hybrid Res-TCN-SE-Attention architecture in which a residual temporal convolutional backbone processes the raw window while 4-level Daubechies-4 DWT statistics (175 features per window) and FEATS vectors are each passed through small two-layer MLP branches and concatenated with the convolutional representation before the final classifier (Section 2.5.1). In the OSW protocol, DWT and FEATS are used either as inputs to FEATS-only classical baselines or as auxiliary branches in the hybrid Res-TCN-SE-Attention model, while RAW-only models provide end-to-end baselines on the same window representation.

For the Res-TCN-SE-Attention model, optimization used the Adam optimizer with binary cross-entropy loss, mini-batches of 128 windows, and a cosine-decay-with-restarts learning-rate schedule; the model was trained for a fixed number of epochs without early stopping, and the final weights were used for evaluation. The other convolutional OSW baselines (shallow 1D CNN, one-dimensional ResNet, InceptionTime1D, DeepConvNet-lite, and EEGNet) also used Adam with binary cross-entropy on RAW windows, but were trained with early stopping on validation AUC (patience ≈ 10 epochs) and restoration of the best weights. Where present, Gaussian noise layers on the input acted as regularization; no additional on-the-fly data augmentation was applied in the subject-dependent regime.

To quantify the contribution of the key architectural modules in the proposed Res-TCN-SE-Attention model, we retrained controlled ablation variants by removing (i) the attention-pooling mechanism, (ii) the squeeze-and-excitation (SE) modulation, or (iii) both modules, while keeping the OSW split, inputs (RAW + DWT + FEATS), and optimization settings unchanged; results are summarized in Section 3.1.

Classical baselines (LR, RF, k-NN, MLP on features, SVM-RBF, and GB) operated exclusively on the FEATS vectors. Features were standardized using training-set statistics; key hyperparameters (e.g., number of trees and maximum depth for tree models, number of neighbors for k-NN, hidden-layer sizes and regularization for MLP, and C for SVM) were chosen from small grids within commonly used ranges. These models were trained on the training windows, optionally tuned on validation windows, and finally assessed on the held-out test windows.

For all subject-dependent models, we first computed window-level posterior probabilities on the validation windows and used Youden’s J statistic on the corresponding ROC curve to select a single decision threshold that maximized the difference between true-positive and false-positive rates for the OSW split. This threshold was then fixed and applied to the test windows. Primary metrics were window-level accuracy, balanced accuracy, precision, recall, F1-score, ROC AUC, Matthews correlation coefficient (MCC), and area under the precision–recall curve (PR AUC). Session-level decisions can be obtained, where desired, by averaging window-level probabilities over all windows from the same recording and applying the same validation-derived threshold; where such session-level results are reported, this aggregation rule is used consistently.

#### 2.6.3. Cross-Subject Training and Evaluation

The subject-independent analyses aimed to estimate generalization to unseen individuals. All cross-subject pipelines enforced that all windows from a given subject appeared either in the training/validation part or in the test part of a given fold, never in both.

For the main ResNet-SE architecture, we used a grouped K-fold cross-validation scheme by subject. ATAR-preprocessed, band-limited EEG was segmented into overlapping 3.0 s windows with a 0.25 s hop (384 samples at 128 Hz), and each window was z-scored channel-wise. A five-fold grouped cross-validation split was applied with subject ID as the grouping variable. Where available, we used scikit-learn’s StratifiedGroupKFold (with shuffling and a fixed random state) to preserve class balance while grouping windows by subject, falling back to GroupKFold otherwise. In each fold, the splitter provided indices for a train + validation pool and a test set; the test set comprised all windows from a subset of subjects, and the remaining subjects contributed windows to the train/validation pool. Within this pool, we reserved approximately 10% of the non-test windows for validation and used the rest for training, so windows from a given non-test subject could appear in both training and validation sets but never in the test set. Each subject served as a test subject in exactly one fold.

ResNet-SE operated on raw windows (RAW) only in the cross-subject experiments (Section 2.5.2). In addition to this main deep-learning baseline, the classical cross-subject baselines operate on the reduced 65-dimensional band-power/Hjorth/ratio feature set (standardized on the training split). To control for potential confounds due to model architecture and input features, we additionally evaluated the Res-TCN-SE-Attention model under the same subject-wise GroupKFold protocol (Table 5). For this controlled cross-subject run, windows were segmented as in Table 5 (2.0 s or 3.0 s, 0.25 s hop), and the corresponding DWT and FEATS vectors were recomputed per window using the same definitions as in Section 2.4. Fold construction and the train/validation split followed the same subject-disjoint procedure described above, and session-level scores were obtained by averaging per-window probabilities within each session before thresholding. Training used the Adam optimizer with binary focal loss to compensate for the modest window-level class imbalance. A data pipeline applied shuffling, light Gaussian noise injection (σ ≈ 0.01) and MixUp augmentation on training batches only; validation and test windows were not augmented. A ReduceLROnPlateau scheduler and model checkpointing monitored the validation ROC AUC and retained, for each fold, the weights achieving the best validation performance.

Other convolutional and spatial-filter pipelines in the cross-subject experiments (ShallowConvNet, InceptionTime-SE, EEGNet++, FBCSP followed by shrinkage LDA, and Riemannian covariance features with tangent-space logistic regression) operated on 4.0 s windows with a 0.5 s hop extracted from the same ATAR-preprocessed recordings, again with per-window z-scoring. These models used a LOSO protocol: in each run, one subject was held out for testing, the remaining subjects formed the training pool, and a small validation subset was drawn from the training subjects. Time-domain augmentations for the CNN baselines consisted of label-preserving operations such as additive noise, moderate amplitude scaling, limited temporal shifts, and simple time masking; where indicated in Table 5, MixUp was also applied within batches. The FBCSP + LDA and Riemannian TS + LR pipelines did not use data augmentation.

Classical cross-subject baselines (k-NN, ExtraTrees, LDA, LR, RF, SVM-RBF, GaussianNB, and boosted trees including LightGBM, CatBoost, GB, and XGBoost) were trained on session-level descriptive feature vectors. For these models, we formed a single session-level descriptive feature vector (DESC, 65 dimensions) by computing the 2.0 s band-power/Hjorth/ratio features described in Section 2.4 for each window and then averaging these 65-dimensional vectors across all windows within a recording. Grouped K-fold cross-validation by subject (10 folds) was used throughout, with standardization fitted on training sessions within each fold. No augmentations were applied in feature space.

For ResNet-SE, we obtained window-level posterior probabilities on the test windows in each fold and aggregated them to the session level by averaging probabilities over all windows from the same (subject, session) pair. After collecting these out-of-fold session-level scores across all folds (each session predicted by a model that never saw its subject during training), we estimated a single global decision threshold using Youden’s J statistic on the ROC curve of these cross-validated scores. The 66.70% session-level accuracy reported for ResNet-SE in Table 5 corresponds to this global threshold. For the remaining models in Table 5, session-level decisions were obtained by applying the default 0.5 cutpoint to the aggregated session probabilities or scores within each fold.

Because short, overlapping windows are strongly autocorrelated, we treat session-level performance as the primary independent unit for inference in the cross-subject setting. This aligns with the operational goal of deciding whether a given recording indicates deception or truth and reduces variance introduced by heavily overlapping window predictions. Where repeated runs are performed (e.g., the subject-dependent ablation study), results are reported as mean ± standard deviation across training seeds under an otherwise fixed protocol. For completeness, we also compute per-window metrics (accuracy, AUC, precision, recall, F1, balanced accuracy, MCC, and PR AUC) and report ROC and precision–recall curves in Section 3.

## 3. Results

Because practical use requires generalization to individuals not seen during training, we treat the cross-subject results in Section 3.2 as the primary indicator of performance. The subject-dependent OSW results in Section 3.1 are reported as an optimistic subject-dependent reference and quantify the size of the generalization gap.

### 3.1. Subject-Dependent Results

**Figure 5:**
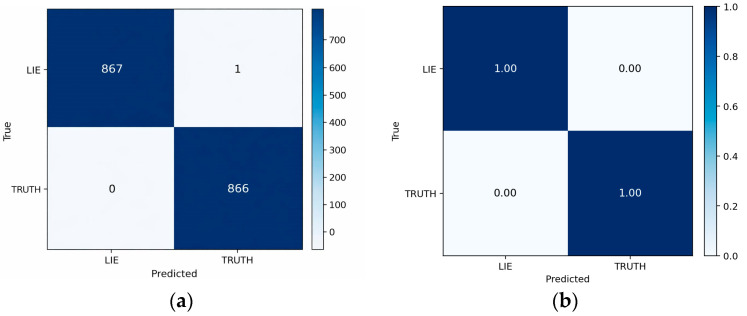
Confusion matrices for the Residual Temporal Convolutional Network with Squeeze-and-Excitation and Attention (Res-TCN-SE-Attention) model on the subject-dependent overlapping sliding-window (OSW) test split: (**a**) window-level confusion matrix (counts; TN = 867, FP = 1, FN = 0, and TP = 866) using the validation-derived threshold $t^{*}$; and (**b**) corresponding normalized confusion matrix (per-class rates), with ≈1.00 on the diagonal.

On the subject-dependent, session-disjoint overlapping sliding-window split (single fixed stratified session-level 80/10/10 train/validation/test partition; 1734 test windows with roughly balanced classes), the hybrid Res-TCN-SE-Attention model with RAW + DWT + FEATS inputs (Section 2.5.1) achieves window-level ACC = 99.94%, AUC = 100.00%, F1 = 99.94%, and macro-averaged precision and recall of 99.94% on the held-out test windows (Table 6). This OSW partition is defined at the recording/session level, and all overlapping windows extracted from a given session inherit its subset assignment; the near-ceiling results therefore reflect a subject-dependent setting with highly autocorrelated overlapping windows rather than within-session train–test overlap. Accordingly, we report these numbers as a protocol-specific ceiling reference and not as evidence of subject-independent deception-discriminative learning. Using class labels 0 = LIE and 1 = TRUTH, the LIE class attains precision = 100.00% and recall ≈ 99.88%, while the TRUTH class attains precision ≈ 99.88% and recall = 100.00%. The confusion matrix in Figure 5 shows only one false positive and no false negatives (TN = 867, FP = 1, FN = 0, and TP = 866), yielding specificity ≈ 99.88%, balanced accuracy ≈ 99.94%, PR-AUC ≈ 100.00%, and MCC ≈ 0.999. The operating threshold selected on the validation windows by Youden’s J was $t^{*}\approx 4\times 10^{-4}$, reflecting highly polarized sigmoid outputs (most window probabilities very close to 0 or 1), so performance is largely insensitive to the precise choice of cutoff within a broad range that includes the conventional 0.5 threshold.

To quantify the contribution of the SE and attention modules in the proposed Res-TCN-SE-Attention architecture under the subject-dependent OSW protocol, we trained four variants (Table 7) using identical preprocessing, the same fixed OSW split, and the same training setup across three random seeds. In this OSW protocol, the 80/10/10 partition is defined at the recording/session level, and all windows extracted from a session inherit its subset assignment. Performance was evaluated at both the window level and the session level (session score obtained by averaging window probabilities within each session), with the decision threshold calibrated on the validation set using Youden’s J. Table 7 reports mean ± SD across seeds. The full model achieved 99.94% window-level accuracy and 100% session-level accuracy in all runs (6/6). Removing SE alone did not change session-level performance (6/6 in all runs), whereas removing attention (replacing attention pooling with global average pooling) introduced a small degradation (mean session ACC 99.38% ± 1.07%; worst seed 5/6; Wilson 95% CI: 90.23–99.67%). Removing both SE and attention produced the largest drop (mean session ACC 98.15% ± 3.21%; worst seed 5/6; Wilson 95% CI: 84.89–98.09%). Overall, all variants remain near-ceiling in the subject-dependent OSW split, which is consistent with the optimistic nature of subject-dependent evaluation on highly overlapping windows; the ablation mainly reflects operating-point stability rather than a decisive change in separability, motivating the protocol-aware cross-subject evaluation in Section 3.2.

Beyond the proposed hybrid architecture, most subject-dependent baselines in Table 4 also achieve very high window-level accuracies (≈90–99%). CNNs trained directly on RAW windows (InceptionTime1D, ResNet1D, DeepConvNet-lite, and a Shallow 1D-CNN) all exceed 97% accuracy, while the EEGNet variant and FEATS-only classical models (RF, k-NN, MLP, SVM-RBF, GB, and LR) obtain between ≈61% and 96%. These results indicate that, under the subject-dependent, session-disjoint OSW setting, a wide range of architectures can exploit the strong within-participant (subject-/session-specific) signal, with the RAW + DWT + FEATS Res-TCN-SE-Attention model providing a small but consistent improvement over RAW-only and FEATS-only alternatives.

**Figure 6:**
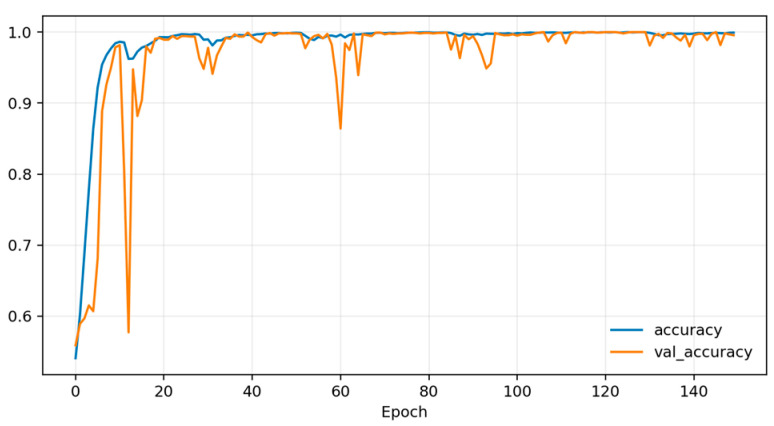
Training and validation accuracy across 150 epochs for the Residual Temporal Convolutional Network with Squeeze-and-Excitation and Attention (Res-TCN-SE-Attention) model on the subject-dependent overlapping sliding-window (OSW) split.

**Figure 7:**
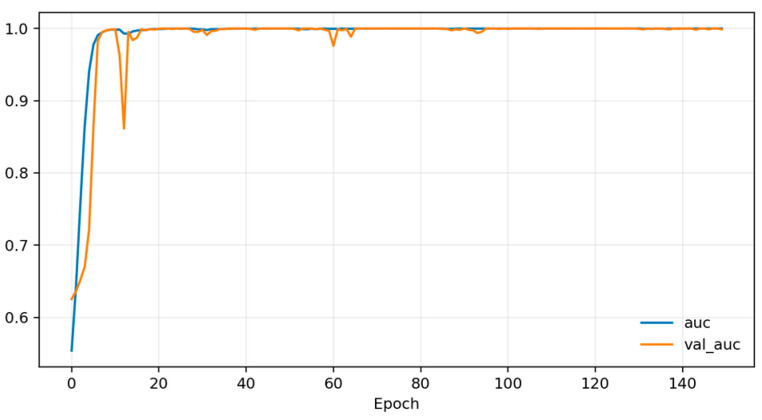
Training and validation ROC AUC across 150 epochs for the Residual Temporal Convolutional Network with Squeeze-and-Excitation and Attention (Res-TCN-SE-Attention) model on the subject-dependent overlapping sliding-window (OSW) split.

**Figure 8:**
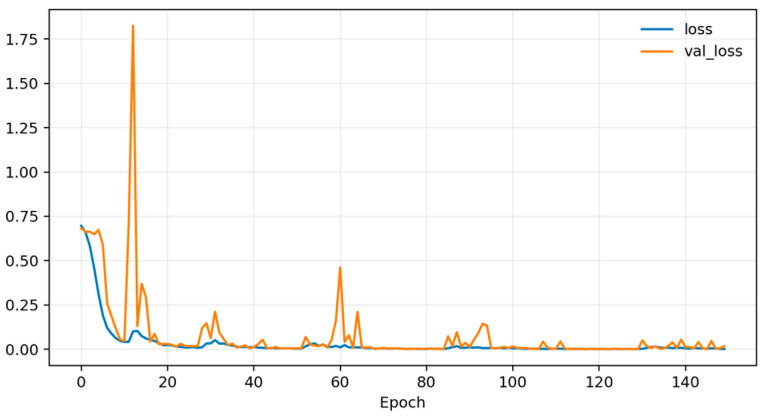
Training and validation loss across 150 epochs for the Residual Temporal Convolutional Network with Squeeze-and-Excitation and Attention (Res-TCN-SE-Attention) model; brief validation spikes coincide with learning-rate restarts and are resolved as training proceeds.

Training dynamics (Figure 6, Figure 7 and Figure 8) indicate rapid convergence and stable behavior on the validation set. Accuracy and AUC exceed 0.98 within roughly the first 10 epochs and then plateau close to 1.0, while the validation loss shows occasional short spikes that coincide with learning-rate restarts and mini-batch stochasticity. Despite these transients, the validation curves quickly realign with the training curves, and no sustained overfitting is observed over the 150 training epochs. Practically, these findings show that, under a single fixed, subject-dependent, session-disjoint split with heavily overlapping windows, the model can almost perfectly separate lie and truth windows from the same pool of individuals. Because this protocol is subject-dependent (the same participants may occur in multiple subsets via different sessions) and the evaluation relies on a single predefined stratified session-level 80/10/10 split, we treat these figures as an optimistic upper bound used mainly to validate modeling choices and threshold/ROC behavior before turning to the more challenging and practically relevant cross-subject setting.

### 3.2. Cross-Subject Results

We assessed subject-independent generalization with five-fold grouped cross-validation by subject using the ResNet-SE model trained on ATAR-preprocessed 3.0 s RAW windows with focal loss and light data augmentation (Section 2.5.2). The network outputs window-level posterior probabilities, which we average within each (subject, session) to obtain a single scalar score per session, the operational unit in practice. Because any deployment would issue one decision per recording, we treat session-level accuracy as the primary endpoint: the short, heavily overlapping windows used during training are intermediate units and are strongly autocorrelated, so window-level metrics can overstate performance and misrepresent the cost of misclassifying an entire session. Aggregating window probabilities within each session and calibrating a single global decision threshold on the pooled cross-validated session scores using Youden’s J (Section 2.6.3) yields a session-level accuracy of 66.70% with session-level AUC = 57.80%, which we report as the deployment-style operating point (Table 8). Class-wise behavior is asymmetric: recall is higher for LIE (74.10%) than for TRUTH (59.30%), whereas precision is slightly higher for TRUTH (69.60%) than for LIE (64.50%). Session-level support is balanced (27 lie and 27 truth sessions; total of 54).

To better quantify the robustness of the cross-subject results in Table 8, we summarize the session-level operating point in terms of the confusion matrix, confidence intervals, and fold-to-fold variability. The session-level accuracy of 66.70% corresponds to 36/54 correctly classified sessions (TRUTH treated as the positive class: TN = 20, FP = 7, FN = 11, and TP = 16), with a 95% Wilson interval of 53.4–77.8%. At this operating point, recall for LIE (class 0; equivalent to specificity under this convention) is 74.1% (20/27; 95% CI 55.3–86.8%), while recall for TRUTH (class 1; sensitivity) is 59.3% (16/27; 95% CI 40.7–75.5%), indicating modest but asymmetric generalization. Fold-wise variability is substantial (session-level AUC: 0.32–0.88; mean ± SD = 0.572 ± 0.201), while window-level accuracy and AUC averaged 0.505 ± 0.025 and 0.545 ± 0.091 across folds, respectively. For completeness, treating sessions as independent (noting that each subject contributes two sessions), an exact two-sided binomial test against chance (*p*_0_ = 0.5) yields *p* = 0.020 on the pooled out-of-fold session decisions.

Table 5 places this result in the context of additional cross-subject baselines. Classical models trained on session-level descriptive features reach between chance level (≈50%) and ≈57% accuracy, with k-NN and ExtraTrees performing best among them (57.40% and 55.60%, respectively). CNN and spatial-filter pipelines evaluated under LOSO on 4.0 s windows (FBCSP + LDA, ShallowConvNet, InceptionTime-SE, Riemann TS + LR, EEGNet++) cluster around 49–53% accuracy. Overall, ResNet-SE on 3.0 s windows provides the highest cross-subject session-level accuracy in our study, but the improvement over chance remains modest, underlining the difficulty of generalizing deception-related patterns from low-channel wearable EEG across individuals.

**Figure 9:**
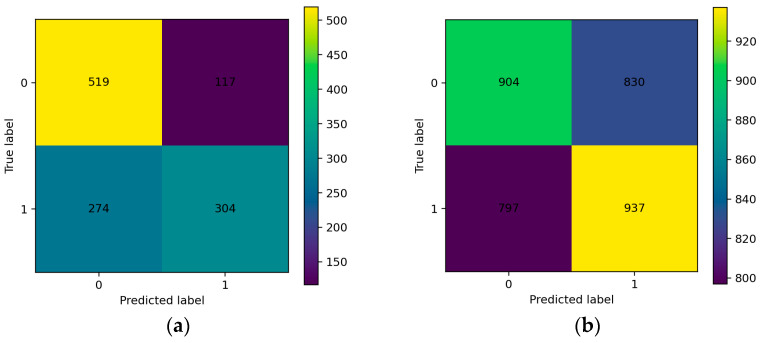
Window-level confusion matrices for the Residual Network with Squeeze-and-Excitation blocks (ResNet-SE) model on the best validation fold (Fold 4): (**a**) validation windows using the fold-specific threshold $t_{\mathrm{val}}\approx 0.315$ selected on the validation ROC curve; and (**b**) test windows from the same fold evaluated with the same threshold.

To illustrate typical window-level behavior, we show results for the fold with the highest validation AUC (Fold 4, validation AUC ≈ 0.79). Within that fold, a window-level decision threshold $t_{\mathrm{val}}\approx 0.315$ is selected from the validation ROC curve; Figure 9a,b depict the corresponding validation and test confusion matrices at the window level, illustrating the per-fold operating point obtained using the validation-derived threshold.

**Figure 10:**
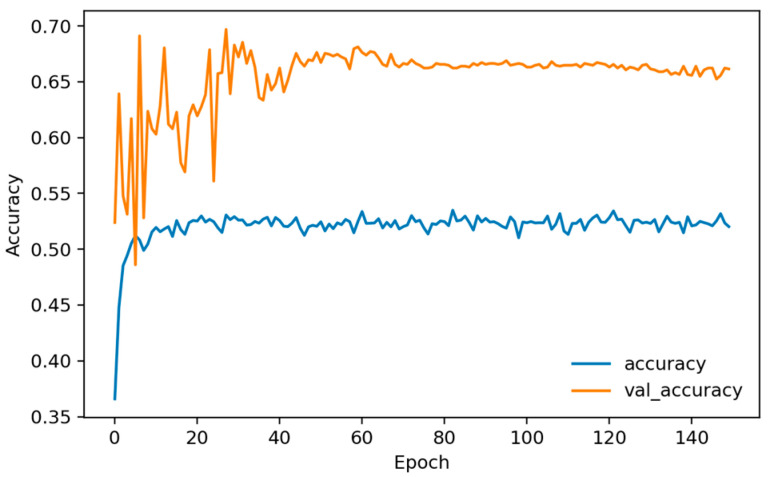
Training and validation accuracy across 150 epochs for the Residual Network with Squeeze-and-Excitation blocks (ResNet-SE) model on Fold 4.

**Figure 11:**
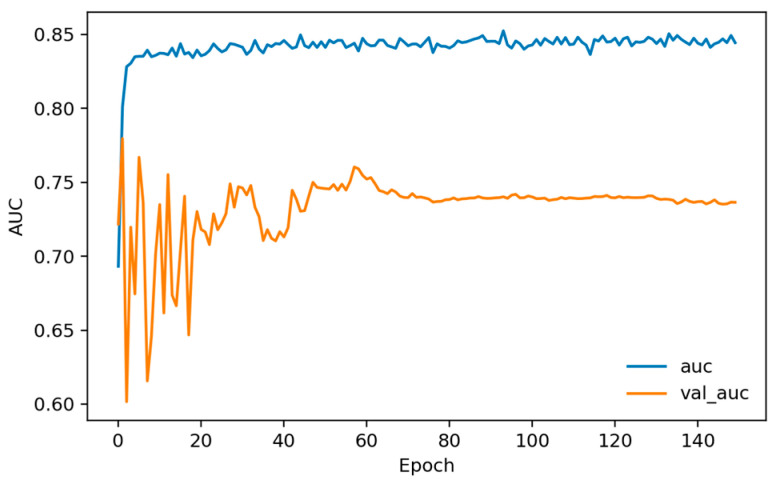
Training and validation ROC AUC across 150 epochs for the Residual Network with Squeeze-and-Excitation blocks (ResNet-SE) model on Fold 4.

**Figure 12:**
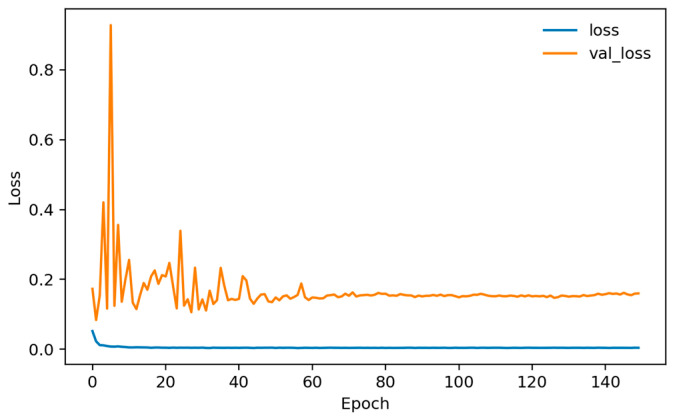
Training and validation loss across 150 epochs for the Residual Network with Squeeze-and-Excitation blocks (ResNet-SE) model on Fold 4.

Figure 10, Figure 11 and Figure 12 plot training and validation accuracy, AUC, and loss over 150 epochs. These curves indicate that the model converges quickly, maintains a validation AUC in the low-to-mid 0.7 range, and does not show dramatic overfitting, even though the absolute cross-subject session-level performance across folds remains moderate.

Taken together, these results show that, even with subject-wise grouped cross-validation, ROC-based calibration, and session-level aggregation, cross-subject performance remains only moderately above chance. Compared to the near-perfect subject-dependent window-level figures in Section 3.1, the ResNet-SE session-level accuracy of 66.70% represents a drop of roughly 30 percentage points, despite the more forgiving aggregation and evaluation protocol. This gap highlights the substantial challenge of generalizing deception-related patterns across individuals from low-channel wearable EEG and motivates a more cautious interpretation and discussion of potential deployment scenarios in Section 4.

### 3.3. Computational Efficiency

Table 9 summarizes the computational footprint and runtime of the two main deep-learning pipelines.

We profiled the computational footprint of the best-performing subject-dependent OSW pipeline (Res-TCN-SE-Attention with RAW + DWT + FEATS; 2.0 s windows, 0.25 s hop). The network has 645,770 parameters (0.646 M) and a 7.68 MB serialized weights file. Training required 13.85 ± 0.30 s/epoch on our workstation (TensorFlow/Keras; NVIDIA RTX A4500 Laptop GPU), corresponding to 2086 s (~34.8 min) over 150 epochs. Model-only inference latency was 5.79 ms/window at batch size 1 and 0.31 ms/window at batch size 64. End-to-end processing of a typical session (293 windows, including preprocessing, feature extraction, inference, and aggregation) required 4.80 s, dominated by DWT/FEATS feature extraction (4.10 s). Peak memory during training was ~1.36 GB RAM and ~2.47 GB GPU memory (device-level used memory).

For the cross-subject CNN–ResNet-SE model (3.0 s windows, 0.25 s hop; five-fold subject-wise grouped cross-validation), the network contains 0.93 M parameters (0.93 M total; 0.928 M trainable). The serialized model occupies 3.78 MB (.keras), while the fold-best checkpoint occupies 10.88 MB (HDF5). Training required 26.45 ± 1.52 s/epoch, corresponding to 3974 ± 230 s per fold (~66.2 ± 3.8 min for 150 epochs). Inference latency was 4.99 ms/window at batch size 1 and 0.63 ms/window at batch size 64. For a median-length session (289 windows), inference plus mean-probability aggregation required 0.63 s (Table 9); including I/O and window construction, end-to-end runtime was ~0.64 s/session. Peak memory during training was 1.45 ± 0.15 GB RAM and ~2.36 ± 0.11 GB GPU memory (device-level used memory).

## 4. Discussion

Our results reveal a pronounced gap between subject-dependent performance and subject-independent generalization. Under the subject-dependent, session-disjoint OSW setting, short overlapping windows carry a very strong discriminative signal and yield near-ceiling figures (≈99.94% accuracy and ≈1.00 AUC at the window level). We primarily use these subject-dependent results as a ceiling check for architecture choices and threshold behavior. When subjects are held out, however, performance drops substantially, underscoring the difficulty of learning a single global decision rule from brief, low-channel wearable EEG segments in the presence of large between-subject variability.

For deployment, session-level accuracy is the relevant endpoint. In practice, a system would issue one decision per (subject, session), whereas short, heavily overlapping windows are only intermediate training units and are strongly autocorrelated, conditions that can inflate window-level metrics and obscure the operational cost of errors. In our subject-independent setting, aggregating window probabilities within each session and calibrating a single global threshold on the pooled cross-validated session scores yields 66.70% session-level accuracy with session-level AUC = 57.80% (Table 8). We report this value as the deployment-style operating point. By contrast, the stricter cross-subject CV statistics reported at the window level, where each fold uses its own validation set for monitoring and model selection and we focus primarily on threshold-free metrics such as AUC, provide our primary, threshold-agnostic estimate of out-of-sample separability for ablations and method comparisons.

The cross-subject regime also exhibits systematic asymmetry between classes. At the calibrated operating point, recall is higher for LIE than for TRUTH, whereas precision is slightly higher for TRUTH (Table 8). This pattern is consistent with confusion matrices from individual folds and corresponds to a relatively conservative operating point that favors avoiding false positives (incorrectly labeling a truth session as deception) at the cost of missing some lies. The best validation fold (Fold 4) selected a substantially lower window-level threshold than the remaining folds and showed smoother training dynamics; we only display this fold’s diagnostics (validation/test window confusion matrices and training curves) for clarity, while all quantitative comparisons use the pooled cross-validated sessions.

Because the subject-independent and subject-dependent pipelines differ not only in evaluation protocol but also in architecture and feature set (ResNet-SE on RAW windows vs. a hybrid Res-TCN-SE-Attention model on RAW + DWT + FEATS), the observed performance gap cannot be attributed solely to the choice of split. Instead, it illustrates how optimistic results can become when subject-dependent, heavily overlapping windowing is combined with a flexible model on the same dataset. Even so, the ≈30-percentage-point drop in accuracy when moving from that setting to cross-subject, session-level evaluation, despite threshold calibration and aggregation, highlights how much of the apparent signal is subject- or session-specific.

To disentangle the effect of evaluation protocol from potential confounds due to model choice and input features, we additionally evaluated (i) ResNet-SE under the subject-dependent OSW split (Table 4) and (ii) the proposed Res-TCN-SE-Attention model with the same RAW + DWT + FEATS input regime under subject-independent GroupKFold by subject (Table 5). Under the OSW setting, both architectures achieve near-ceiling window-level performance, whereas under cross-subject evaluation, the same models drop to moderate or near-chance session-level accuracy. Taken together, these controlled comparisons indicate that the dominant driver of the observed gap is the subject-independent transfer difficulty induced by the evaluation protocol, while architecture and feature choices mainly modulate the absolute level of cross-subject performance.

Why does subject-independent AUC remain modest? Several factors are likely at play. First, EEG exhibits substantial inter-subject non-stationarity: anatomy, exact sensor placement, impedance, habitual muscle artifacts, and individual cognitive strategies all shift the distribution of signals in ways that short-window models cannot fully align without explicit normalization or alignment. Second, the labels in deception paradigms capture a mixture of processes—task difficulty, emotional load, attention, motor preparation, and arousal—alongside deception itself. If these factors differ systematically between conditions, models may learn confounds rather than deception-specific signatures, and such confounds need not generalize across people. Third, the combination of a limited number of subjects and relatively expressive deep architectures places us in a small-sample deep-learning regime: the networks can become excellent subject-dependent recognizers while remaining only moderately effective subject-independent classifiers. In this context, simply adding more windows from the same individuals is not sufficient; diversity in subjects, paradigms, and recording conditions is more important than raw sample count.

Placed in the context of prior EEG-based deception work, our findings align with the broader observation that high accuracies are common under subject-dependent or mixed splits, whereas true subject-independent results are markedly lower once overlap, per-subject normalization, and threshold selection are controlled. Table 10 summarizes a non-exhaustive set of representative studies, explicitly separating subject-dependent and subject-independent evaluation.

Within this framing, our 66.70% calibrated session-level accuracy on LieWaves is in line with recent cross-subject reports that emphasize deployment-style evaluation on low-channel or wearable-style EEG, while our subject-dependent figures resemble the near-perfect performances often reported under subject-dependent or pooled-window protocols. The comparison also underscores the limits of cross-study benchmarking: datasets differ in hardware, montage, stakes, and task design, and many works do not fully specify their splitting and thresholding rules.

Methodologically, the present study deliberately favored a minimal preprocessing pipeline and compact architectures to keep the path to translation simple. This improves reproducibility and makes the constraints of low-channel, wearable EEG explicit, but likely leaves performance on the table in the cross-subject setting, where distribution shift dominates. Several avenues appear promising: (i) Subject alignment and normalization strategies (e.g., per-subject baselines, instance or layer normalization tuned per subject, covariance-space or Riemannian alignment, or domain-adversarial objectives) could reduce inter-subject drift before or within deep models; (ii) longer-horizon modeling that aggregates information over tens of seconds or entire sessions, for example, through temporal pooling, recurrent layers, or transformers, may capture deception strategies and control-related processes that are not visible in isolated 2.0–4.0 s windows; and (iii) explicit probability calibration (e.g., temperature scaling or isotonic regression) could stabilize threshold selection across folds, cohorts, and sites, and make session-level scores more interpretable for downstream decision support.

In addition to evaluation protocol and representation learning, an important practical consideration for low-channel wearable EEG is robustness to channel loss, noise, or intermittent electrode contact. Because our baseline comparisons are reported under a fixed five-channel montage, we do not yet quantify how performance degrades when individual channels are removed or corrupted. A complementary analysis for future work is therefore a leave-one-channel-out sensitivity study (and corresponding channel-drop/noise stress tests) under the same cross-subject protocol, to rank the relative contribution of each channel and to characterize the stability of the session-level decision rule under partial channel availability.

A key limitation of our work, shared by most public deception datasets, is ecological validity. LieWaves and similar corpora induce instructed lying under relatively low or moderate stakes. If motivational salience and arousal modulation are important mediators of deception-related EEG dynamics, then higher-stakes paradigms could amplify separable patterns (e.g., frontal alpha/theta changes and fronto-parietal control rhythms), especially at the session level, where decisions reflect sustained behavior rather than isolated transients. Sample sizes are also modest (27 participants; 54 total sessions, each ~75 s), which limits the precision of cross-subject estimates and contributes to substantial fold-to-fold variability; accordingly, generalization claims should be treated as preliminary until validated on larger cohorts, longer recordings, and independent acquisition conditions. In particular, additional subjects and recordings collected across days/sites are needed to assess robustness to inter-individual and acquisition variability.

In summary, this study clarifies what can, and cannot, currently be expected from short-window, five-channel wearable EEG for deception detection in a subject-independent setting. The cross-subject results demonstrate that there is a non-trivial deception-related signal that generalizes across people under realistic hardware constraints, but they also show that performance remains only moderately above chance once evaluation is aligned with operational decision-making. Transparent reporting of windowing, splitting, aggregation, and threshold calibration is therefore essential for an honest appraisal of readiness. The 66.70% deployment-style operating point, paired with a strict cross-subject CV as the generalization anchor, provides a clear and reproducible baseline on which to test subject-alignment methods, longer-context models, and, critically, improved data collection with meaningful stakes.

## 5. Conclusions

We investigated EEG-based deception detection from short, overlapping windows recorded with a five-channel, wearable head-mounted montage (LieWaves dataset) under both subject-dependent and subject-independent protocols. We examined two complementary settings: (i) a subject-dependent, session-disjoint regime (participants may appear in multiple subsets via different sessions) used as an optimistic ceiling, and (ii) a subject-independent regime evaluated both with strict cross-subject cross-validation and with a calibrated session-level operating point.

When subjects were held out, a compact ResNet-SE trained on ATAR-preprocessed 3.0 s windows with MixUp augmentation and focal loss yielded only modest window-level generalization, but a single global threshold calibrated on cross-validated session scores recovered a deployment-style operating point of 66.70% session-level accuracy with AUC = 57.80%.

In the subject-dependent OSW configuration, a hybrid Res-TCN-SE-Attention model with RAW + DWT + FEATS inputs achieved near-ceiling window-level performance (ACC = 99.94%, AUC ≈ 1.00; one false positive and no false negatives), showing that, under this subject-dependent OSW setup, highly overlapping short windows are strongly autocorrelated and therefore highly separable within the same pool of individuals; however, this separability reflects an optimistic, protocol-specific regime and does not constitute evidence of deployment-relevant deception detection.

Because real systems would issue one decision per (subject, session), we argue that such session-level metrics, together with an explicit statement of whether calibration on target data is allowed, are essential for honest reporting; strict cross-subject CV statistics (primarily AUC and related threshold-free measures) should remain the primary baseline for method comparisons and ablations.

Overall, the study provides a transparent, fully specified baseline and clarifies the gap between optimistic subject-dependent performance and realistically evaluated subject-independent performance on low-channel wearable EEG. The results show that there is a non-trivial deception-related signal that generalizes across people under constrained hardware, but also that performance remains only moderately above chance once evaluation is aligned with operational decision-making. Closing this gap will likely require subject-alignment strategies to mitigate inter-subject non-stationarities, longer-horizon modeling that aggregates information beyond short 2.0–4.0 s windows into tens-of-seconds or session-level context, and improved paradigms and datasets with meaningful stakes and harmonized evaluation protocols.

## Figures and Tables

**Table 1:** Public EEG datasets containing deception-related paradigms considered in this study.

Dataset	Ch	f_s_ (Hz)	N	Paradigm	Access
LieWaves [62]	5	128	27	Two sessions per subject: truth-telling/deception; standardized visual stimuli; and raw CSV	Public
Deception_data [63]	128	3000	44	Competitive two-player deception card game; spontaneous truthful/false claims; rich trial taxonomy	Public
Bag-of-Lies [64]	13	128	35	Naturalistic deception recordings; multimodal (video, audio, and gaze)	By request
Dryad [65]	14	500	34	Concealed-information-style ERP (guilty/innocent groups); P300 analyses	Public

**Table 2:** Quartiles (Q1, median, Q2, Q3) and IQR of the cross-channel mean band power by class (TRUTH vs. LIE). $∆M_{t-1}$ denotes the difference in medians (TRUTH–LIE).

Band	Q1_t_	Q1_l_	M_t_	M_l_	Q3_t_	Q3_l_	IQR_t_	IQR_l_	$∆M_{t-1}$
alpha	17.679	19.453	27.473	30.704	48.440	51.594	30.761	32.140	−3.231
beta	28.649	27.363	42.351	38.539	62.631	64.630	33.983	37.267	+3.811
delta	72.241	97.633	281.562	305.294	894.176	1007.889	821.935	910.257	−23.733
gamma	10.691	10.902	17.573	15.840	31.272	28.100	20.581	17.198	+1.733
theta	24.882	31.199	60.025	78.128	197.725	224.502	172.843	193.303	−18.103

Note: Subscripts t and l denote TRUTH and LIE, respectively; M denotes the median.

**Table 3:** Cliff’s Δ effect size (TRUTH–LIE) for the cross-channel mean band power in each band; all magnitudes are ≤0.088, indicating very small between-class effects.

Band	$∆{Cliff}_{t-1}$
alpha	−0.064
beta	+0.034
delta	−0.066
gamma	+0.038
theta	−0.088

**Table 4:** Subject-dependent models. Unless otherwise noted, models were trained and evaluated using overlapping 2.0 s windows with a 0.25 s hop at 128 Hz extracted from the LieWaves recordings. Sessions were first partitioned using a single stratified 80/10/10 recording/session-level split (all windows from a session inherit its subset assignment), and the same fixed partition was reused across all subject-dependent models for comparability. Reported accuracies are at the window level.

Model	Input Regime	Augmentations (Training Only)	Configuration Summary	Window-Level ACC (%)
Res-TCN-SE-Attention	RAW + DWT + FEATS	Additive Gaussian noise layer on raw input (σ ≈ 0.01)	Residual depthwise-separable temporal CNN with SE and attention pooling on 2.0 s windows; RAW branch fused with 4-level db4 DWT (175-dim) and FEATS (167-dim) vectors, each projected by a 2-layer MLP; Adam optimizer with cosine-decay-with-restarts schedule, binary cross-entropy, and batch size 128	99.94
ResNet-SE *	RAW	Additive Gaussian noise (σ ≈ 0.01), MixUp in mini-batches	Residual 1D CNN with three Conv1D–batch-norm–ReLU–SE stages, global average pooling and dense sigmoid output; trained on 3.0 s windows with Adam, binary focal loss, and batch size 64	99.87
ResNet-SE	RAW	Additive Gaussian noise (σ ≈ 0.01), MixUp in mini-batches	Residual 1D CNN with three Conv1D–batch-norm–ReLU–SE stages, global average pooling and dense sigmoid output; trained on 2.0 s windows with Adam, binary focal loss, and batch size 64	99.50
InceptionTime1D	RAW	none	InceptionTime-style 1D CNN on 2.0 s raw windows (multi-kernel inception blocks with residual shortcuts, global average pooling, and dense sigmoid head); Adam with cosine-decay, binary cross-entropy	99.81
Shallow 1D-CNN	RAW	none	Two Conv1D–batch-norm–ReLU–max-pool blocks on 2.0 s windows, followed by dropout and a dense sigmoid classifier; Adam, binary cross-entropy	99.75
ResNet1D	RAW	none	Three 1D residual Conv1D blocks with batch normalization and ReLU, global average pooling, and dense sigmoid output; Adam with cosine-decay, binary cross-entropy	99.31
DeepConvNet-lite	RAW	none	Compact DeepConvNet-style stack (four temporal Conv1D blocks with batch norm, ELU, pooling, and dropout) followed by dense sigmoid classifier; Adam, and binary cross-entropy	97.85
EEGNet	RAW	none	EEGNet architecture adapted to 5 channels at 128 Hz (temporal, depthwise, and separable convolutions with pooling and dropout, dense sigmoid output); Adam, binary cross-entropy	90.59
RF	FEATS	none	RandomForestClassifier on standardized FEATS (167-dim spectral–statistical + asymmetry vector) with several hundred trees and moderate depth, class-balanced subsampling	96.08
k-NN	FEATS	none	Distance-weighted Euclidean k-NN (k = 7) on standardized FEATS	92.17
MLP	FEATS	none	Two-layer feed-forward network on standardized FEATS (≈256 and 128 ReLU units with dropout and l_2_ regularization), sigmoid output, Adam, and binary cross-entropy	90.71
SVM-RBF	FEATS	none	RBF-kernel SVM on standardized FEATS with C and γ selected from a small grid; class_weight = “balanced”	88.31
GB	FEATS	none	GradientBoostingClassifier on standardized FEATS with a few hundred shallow trees and learning rate ≈ 0.05	84.52
LR	FEATS	none	L_2_-regularized logistic regression on standardized FEATS (solver “lbfgs”, class_weight = “balanced”)	61.59

* ResNet-SE was also trained on 3.0 s windows to match the cross-subject setting.

**Table 5:** Subject-independent (cross-subject) models. All models enforce disjoint training and test subjects using either grouped CV by subject or a leave-one-subject-out (LOSO) protocol. Reported accuracies are at the session level; for window-based models, session scores are obtained by averaging per-window probabilities before thresholding. For the Residual Temporal Convolutional Network with Squeeze-and-Excitation and Attention (Res-TCN-SE-Attention) cross-subject entries, DWT and FEATS were recomputed for the listed window length (2.0 s or 3.0 s), and the model was trained/evaluated under the same GroupKFold-by-subject protocol.

Model	Input	Window/Hop (s)	CV Scheme	Augmentations (Training Only)	Configuration Summary	Session-Level ACC (%)
ResNet-SE	RAW	3.0/0.25	GroupKFold (5 folds, by subject)	Additive Gaussian noise (σ ≈ 0.01), MixUp in mini-batches	Residual 1D CNN with three Conv1D–batch-norm–ReLU–SE stages, global average pooling and dense sigmoid output; trained on 3.0 s windows with Adam, binary focal loss, and batch size 64	66.70
Res-TCN-SE-Attention	RAW + DWT + FEATS	3.0/0.25	GroupKFold (5 folds, by subject)	Additive Gaussian noise layer on raw input (σ ≈ 0.01)	Residual depthwise-separable temporal CNN with SE and attention pooling on 3.0 s windows; RAW branch fused with 4-level db4 DWT (175-dim) and FEATS (167-dim) vectors, each projected by a 2-layer MLP; Adam optimizer with cosine-decay-with-restarts schedule, binary cross-entropy, and batch size 128	54.00
Res-TCN-SE-Attention	RAW + DWT + FEATS	2.0/0.25	GroupKFold (5 folds, by subject)	Additive Gaussian noise layer on raw input (σ ≈ 0.01)	Residual depthwise-separable temporal CNN with SE and attention pooling on 2.0 s windows; RAW branch fused with 4-level db4 DWT (175-dim) and FEATS (167-dim) vectors, each projected by a 2-layer MLP; Adam optimizer with cosine-decay-with-restarts schedule, binary cross-entropy, and batch size 128	49.67
k-NN	DESC	—	GroupK-Fold (10 folds, by subject)	none	Euclidean k-NN (k = 5, distance weighting) on standardized session-level DESC features	57.40
ExtraTrees	DESC	—	GroupK-Fold (10 folds, by subject)	none	ExtraTreesClassifier on standardized session-level DESC features with several hundred extremely randomized trees and shallow depth	55.60
FBCSP + LDA	RAW	4.0/0.5	LOSO	none	Filter-bank CSP features from multiple 1–45 Hz sub-bands computed on 4.0 s windows, concatenated and classified with shrinkage LDA in a leave-one-subject-out setup	52.80
ShallowConvNet	RAW (+MixUp)	4.0/0.5	LOSO	Gaussian noise, mild amplitude scaling, MixUp	ShallowConvNet-style CNN on 4.0 s windows (temporal Conv1D blocks with pooling and dense sigmoid head); Adam, binary cross-entropy; LOSO by subject	52.60
InceptionTime-SE	RAW	4.0/0.5	LOSO	Gaussian noise, small time shifts, MixUp	InceptionTime 1D CNN with squeeze-and-excitation blocks on 4.0 s windows; Adam optimizer, binary cross-entropy; LOSO by subject	52.50
LDA	DESC	—	GroupK-Fold (10 folds, by subject)	none	Standard LDA on standardized session-level DESC features with class priors and covariance estimated per fold	51.90
LR	DESC	—	GroupK-Fold (10 folds, by subject)	none	L_2_-regularized logistic regression on standardized session-level DESC features (solver “lbfgs”, class_weight = “balanced”)	51.90
RF	DESC	—	GroupK-Fold (10 folds, by subject)	none	RandomForestClassifier on standardized session-level DESC features with several hundred trees and moderate depth	50.00
SVW-RBF	DESC	—	GroupK-Fold (10 folds, by subject)	none	RBF-kernel SVM on standardized session-level DESC features with C and γ chosen from a small grid; probabilities from Platt scaling	50.00
GaussianNB	DESC	—	GroupK-Fold (10 folds, by subject)	none	Gaussian Naive Bayes classifier on standardized session-level DESC features with per-feature Gaussian assumptions	50.00
Riemann TS + LR	RAW	4.0/0.5	LOSO	none	Window-wise covariance matrices from 4.0 s segments mapped to Riemannian tangent space and classified with logistic regression; LOSO by subject	49.60
EEGNet++	RAW (+MixUp)	4.0/0.5	LOSO	Gaussian noise, random channel drop, MixUp	EEGNet CNN on raw 4.0 s windows (temporal, depthwise, and separable convolutions with pooling and dropout, dense sigmoid output); Adam, binary cross-entropy; LOSO by subject	49.10
LightGBM	DESC	—	GroupK-Fold (10 folds, by subject)	none	LightGBM on standardized session-level DESC features with tuned numbers of leaves/trees and learning rate in a small grid	48.10
CatBoost	DESC	—	GroupK-Fold (10 folds, by subject)	none	CatBoostClassifier on standardized session-level DESC features with default-depth trees and tuned learning rate	48.10
GB	DESC	—	GroupK-Fold (10 folds, by subject)	none	GradientBoostingClassifier on standardized session-level DESC features with ≈300 shallow trees and moderate learning rate	48.10
XGBoost	DESC	—	GroupK-Fold (10 folds, by subject)	none	XGBoost on standardized session-level DESC features with a few hundred trees and tuned depth/learning rate from a small grid	48.10
Linear SVM	DESC	—	GroupK-Fold (10 folds, by subject)	none	Linear SVM on standardized session-level DESC features with L_2_ regularization parameter C selected from a small grid; class_weight = “balanced”	46.30

Note: DWT/FEATS were recomputed for each window length, and GroupKFold was performed by subject.

**Table 6:** Subject-dependent window-level test results for the Residual Temporal Convolutional Network with Squeeze-and-Excitation and Attention (Res-TCN-SE-Attention) model (RAW + DWT + FEATS inputs).

	Precision (%)	Recall (%)	F1-Score (%)	Support
0	100	99.87	99.94	868
1	99.87	100	99.94	866
accuracy			99.94	1734
macro avg.	99.94	99.94	99.94	1734
weighted avg.	99.94	99.94	99.94	1734

Note: Class labels: 0 = LIE, and 1 = TRUTH. Operating threshold $t^{*}$ estimated on validation windows using Youden’s J (≈0.0004).

**Table 7:** Subject-dependent overlapping sliding-window (OSW) ablation of squeeze-and-excitation (SE) and attention modules (mean ± SD across 3 seeds) under a session-level 80/10/10 split (train/val/test; session-disjoint—no session contributes windows to more than one subset). Window-level metrics are computed over all test windows from the held-out test sessions. Session-level metrics are computed by averaging window posterior probabilities within each session and applying a single decision threshold calibrated on the validation sessions using Youden’s J. “Minimum across seeds” indicates the lowest number of correctly classified test sessions (out of 6) across the three seeds.

Model	Window ACC (%)	Window MCC	Session ACC (%)	Session MCC	Minimum Across Seeds (Sessions Correct)
Res-TCN-SE-Attention	99.94 ± 0.00	0.999 ± 0.000	100.00 ± 0.00	1.000 ± 0.000	6/6
Res-TCN-SE	99.77 ± 0.10	0.995 ± 0.002	99.38 ± 1.07	0.988 ± 0.021	5/6
Res-TCN-Attention	99.77 ± 0.13	0.995 ± 0.003	100.00 ± 0.00	1.000 ± 0.000	6/6
Res-TCN	99.81 ± 0.13	0.996 ± 0.003	98.15 ± 3.21	0.965 ± 0.061	5/6

**Table 8:** Cross-subject session-level results for the Residual Network with Squeeze-and-Excitation blocks (ResNet-SE) model.

	Precision (%)	Recall (%)	F1-Score (%)	Support
0	64.50	74.10	69.00	27
1	69.60	59.30	64.00	27
accuracy			66.70	54
macro avg.	67.00	66.70	66.50	54
weighted avg.	67.00	66.70	66.50	54

Note: Class labels: 0 = LIE, 1 = TRUTH. Metrics are computed on pooled test sessions across the five folds after a single global threshold calibration on session scores using Youden’s J ($t^{*}\approx 0.625$). AUC is computed from the same session-level scores (AUC = 57.80%).

**Table 9:** Computational efficiency and model footprint of the two main pipelines (FP32). Inference latency is reported per window for batch sizes 1 and 64; epoch times are measured after warm-up (epochs ≥ 5). “Session runtime” reflects a single ~75 s LieWaves session; for the OSW hybrid pipeline, it includes preprocessing, feature extraction, inference, and aggregation, whereas for the cross-subject pipeline, it includes inference and mean aggregation only (windows precomputed offline).

	Res-TCN-SE-Attention (Subject-Dependent OSW; RAW + DWT + FEATS)	ResNet-SE (Cross-Subject; GroupKFold)
Parameters (trainable/total)	0.646 M/0.646 M	0.928 M/0.930 M
Weights size	7.68 MB	3.78 MB (.keras)/10.88 MB (.h5)
Training time (s/epoch)	13.846 ± 0.304	26.453 ± 1.520
Total training time	2085.85 s (34.8 min)	3974 ± 230 s/fold (66.2 ± 3.8 min)
Peak RAM	1.36 GB	1.45 ± 0.15 GB
Peak GPU memory	2.47 GB *	2.36 ± 0.11 GB *
Inference (ms/window) batch size = 1	5.793	4.991
Inference (ms/window) batch size = 64	0.311	0.631
Session runtime (s/session)	4.804 (293 windows)	0.631 (289 windows) **

* GPU memory values reflect approximate device-level “used” memory (e.g., via nvidia-smi), not allocator-level per-process usage. ** Session runtime excludes preprocessing/windowing (windows precomputed offline).

**Table 10:** Representative EEG-based deception-detection studies with subject-dependent and subject-independent evaluation protocols.

Reference	Dataset	Classifiers	Accuracy (%)
(**a**) Subject-dependent			
Our method	LieWaves	Res-TCN-SE-Attention (RAW + DWT + FEATS)	99.94
[62]	LieWaves	LSTM + DWT	99.88
[43]	Proprietary dataset	DGCN + Type-2 Fuzzy	~98.00
[44]	Bag-of-Lies	LSTM + NCP	97.88
[45]	Bag-of-Lies	Bi-LSTM	97.00
[70]	Bag-of-Lies	DCNN	95.91
[71]	Proprietary dataset	ICA + SVM	~90.00
	(**b**) Subject-independent (cross-subject)		
Our method	LieWaves	ResNet-SE	66.70
[72]	LieWaves	RBF-SVM	64.07
[64]	Bag-of-Lies	RF	58.71
[73]	Proprietary dataset	XGBoost	53.00

## Data Availability

No new human data were collected for this study. All experiments used the publicly available LieWaves dataset described in Section 2. The original dataset can be obtained from Mendeley Data (dataset ID 5gzxb2bzs2, Version 2): https://data.mendeley.com/datasets/5gzxb2bzs2/2 (accessed on 9 June 2025), https://doi.org/10.17632/5gzxb2bzs2.2. The analysis scripts/notebooks and code used to generate Figures and Tables are available from the corresponding author upon reasonable request, subject to an informal data-sharing agreement. We can also provide de-identified derived artifacts (e.g., window-level feature matrices), the exact subject-wise train/validation/test splits, and aggregate evaluation outputs needed to reproduce the reported results.

## Abbreviations

The following abbreviations are used in this manuscript:

EEG	Electroencephalography
ResNet-SE	Residual Network with Squeeze-and-Excitation blocks
AUC	Area under the curve
Res-TCN-SE-Attention	Residual Temporal Convolutional Network with Squeeze-and-Excitation and Attention
DWT	Discrete wavelet transform
OSW	Overlapping sliding window
ERP	Event-related potential
CIT	Concealed information test
CQT	Comparison question test
CNN	Convolutional neural network
MI	Mutual information
NMI	Normalized mutual information
RBF-ELM	Radial-Basis-Function Extreme Learning Machine
RNN	Recurrent neural network
GNN	Graph neural network
P300	Event-related potential component
CV	Cross-validation
ROC	Receiver operating characteristic
FEATS	The 167-dimensional spectral–statistical feature vector
ATAR	Automatic wavelet-based artifact reduction
ICA	Independent component analysis
LOSO	Leave-one-subject-out
ShallowConvNet	Shallow convolutional network
InceptionTime-SE	InceptionTime network with squeeze-and-excitation blocks
EEGNet++	EEGNet model with multi-band temporal kernels and attention
FBCSP	Filter-bank common spatial patterns
LDA	Linear discriminant analysis
TS	Tangent space
LR	Logistic regression
IIR	Infinite impulse response
PSD	Power spectral density
BCI	Brain–computer interface
SVM	Support vector machine
RF	Random forest
IQR	Interquartile range
MLP	Multi-layer perceptron
ResNet1D	One-dimensional residual network
InceptionTime1D	InceptionTime 1D convolutional network
DeepConvNet-lite	Lightweight deep convolutional network
EEGNet	EEG-specific convolutional neural network
k-NN	k-nearest neighbors
GB	Gradient boosting
SVM-RBF	Support vector machine radial basis function
QDA	Quadratic discriminant analysis
GaussianNB	Gaussian Naive Bayes
XGBoost	Extreme gradient boosting
LightGBM	Light gradient boosting machine
CatBoost	Category boosting
MCC	Matthews correlation coefficient
PR	Precision–recall
DESC	Single session-level descriptive feature vector (65 dimensions)
TN	True negative
FP	False positive
FN	False negative
TP	True positive
SD	Standard deviation
